# Analysis of cellular kinetic models suggest that physiologically based model parameters may be inherently, practically unidentifiable

**DOI:** 10.1007/s10928-022-09819-7

**Published:** 2022-08-06

**Authors:** Liam V. Brown, Mark C. Coles, Mark McConnell, Alexander V. Ratushny, Eamonn A. Gaffney

**Affiliations:** 1 Wolfson Centre for Mathematical Biology, Mathematical Institute, University of Oxford, Oxford, UK; 2 Kennedy Institute of Rheumatology, University of Oxford, Oxford, UK; 3 Bristol Myers Squibb, Seattle, WA USA; 4 Currently Chinook Therapeutics, Seattle, WA USA

**Keywords:** Parameter, Identifiability, Uncertainty

## Abstract

Physiologically-based pharmacokinetic and cellular kinetic models are used extensively to predict concentration profiles of drugs or adoptively transferred cells in patients and laboratory animals. Models are fit to data by the numerical optimisation of appropriate parameter values. When quantities such as the area under the curve are all that is desired, only a close qualitative fit to data is required. When the biological interpretation of the model that produced the fit is important, an assessment of uncertainties is often also warranted. Often, a goal of fitting PBPK models to data is to estimate parameter values, which can then be used to assess characteristics of the fit system or applied to inform new modelling efforts and extrapolation, to inform a prediction under new conditions. However, the parameters that yield a particular model output may not necessarily be unique, in which case the parameters are said to be unidentifiable. We show that the parameters in three published physiologically-based pharmacokinetic models are practically (deterministically) unidentifiable and that it is challenging to assess the associated parameter uncertainty with simple curve fitting techniques. This result could affect many physiologically-based pharmacokinetic models, and we advocate more widespread use of thorough techniques and analyses to address these issues, such as established Markov Chain Monte Carlo and Bayesian methodologies. Greater handling and reporting of uncertainty and identifiability of fit parameters would directly and positively impact interpretation and translation for physiologically-based model applications, enhancing their capacity to inform new model development efforts and extrapolation in support of future clinical decision-making.

**Supplementary Information:**

The online version contains supplementary material available at 10.1007/s10928-022-09819-7.

## Introduction

Physiologically-based pharmacokinetic (PBPK) models are mathematical models that describe the kinetics and dynamics of a transferred cell population (or a drug) in an organism. Biokinetic modelling has existed since at least 1937 [1], when Teorell used physiological data to model the kinetics of substances between the blood and various organ compartments. Since then, these models have been used in numerous studies to simulate the biodistribution of drugs (for recent reviews, see [2–4]). Such calculations are important for drug discovery, allowing the calculation of metrics such as the total drug exposure in different patient populations, and they are accepted and expected as supporting information for clinical trials by both the US Food and Drug Administration and the European Medicines Agency. More recently, PBPK models have been generalised to describe the movement of cells in the body, to understand T-cell trafficking and to predict their localisation [5–11], or their interaction with solid and haematological cancers [12–15]. These models typically consist of many compartments describing vascular, interstitial and other spaces within various organs and tissues of interest. The models require parameters that quantify the rates of processes such as entry and exit from each compartment. Fitting models to data requires optimisation of these parameters. It is usually possible to fit equations closely to data and provide a corresponding set of best fit parameters, but it is much harder to determine whether the solution is unique or to quantify the uncertainty on fit parameters. Simple curve-fitting or non-linear least squares techniques provide the local covariance of the solution at the fit, but this is not a substitute for parameter uncertainty and does not reveal information about the existence of multiple solutions with a similar fit, or about parameter identifiability. We conducted a brief literature survey, selecting some of the most cited references containing the term “PBPK” and published since 2010. Of 13 selected studies [6–11, 14, 16–21], 9 of them used regression (giving no estimates of parameter uncertainty), 3 used Monolix or Adapt V (which can give estimates of uncertainty and indications of identifiability issues through expectation-maximisation, though no author commented on identifiability), and 1 used an unspecified Markov Chain Monte Carlo technique, but published no analysis of parameter uncertainty or identifiability. For many intended applications, such as the calculation of the area under a curve, a close fit to data is sufficient. A set of cubic splines can be mapped to data and a mathematical model of the underlying biology is not required. However, PBPK is often expected to provide supporting evidence to regulatory bodies, for which understanding of the biology and how it relates to observable outputs is desirable. To be able to trust models and extrapolate their results, it is critical to understand their behaviour, uncertainty and limitations. In particular, it is important that estimated parameter values are accurate or that their uncertainty is well-understood, if these parameters correspond to important biological processes such as drug clearance rates. In some cases, this understanding is essential and it is insufficient to obtain a “good” fit to data, typically defined by a low scoring distance metric or a qualitatively close match between data and model output. For example, when scaling model fits to other populations, such as from an adult population to a paediatric population or from a mouse model to humans, one must understand which parameters would be expected to change and must have accurate estimates of those parameters, as a 50% change to an “incorrect” value of a parameter may result in very different predictions to a 50% change to a “correct” value. Another example is when modelling an intervention that would be expected to change a parameter value but where data are not available, for example, as a reduction in egress rates from a site of interest. The uncertainty in the change in an observable outcome such as lesion sizes at a clinical endpoint would be related to the uncertainty in parameter values. That uncertainty can be determined by error propagation or by collective fitting of many parameter sets that could be consistent with available data. Notably, in the latter case, output uncertainty can be constrained if the uncertainty in *groups* of parameters is constrained, even if individual parameters are uncertain [22]. A final example is when two different models of the same biological system have parameters that describe the same processes but with very different values. Without analysing the model construction or parameter fitting processes, one cannot know whether the different parameter values are due to model assumptions or the existence of multiple parameter sets that give similar model outputs.

Most published PBPK modelling studies use techniques that yield *a* fit and report that single fit. Parameter uncertainty or the existence of multiple parameter sets with similar outputs are rarely reported. In this study, we illustrate how these problems manifest in physiologically-based modelling with three different data sets and three different published models [5, 10, 14]. In each case, we produced or used a “good” (close) fit to data, and used synthetic data generated from the fit parameters to show that “good fits” may be misleading for some cases, as original parameters are not necessarily recovered; parameters are not necessarily identifiable. Even if accurate parameter values are not required for a given reported study, published data or parameter values are often utilised by other mathematical modellers, and this may cause unknown uncertainty to be carried forward into a new context. For each of the three models considered here, we show how simple analyses can indicate which parameters may be uncertain after a “good” simple fit is obtained, and under which conditions this will occur for a given parameter. We then show how selected Bayesian techniques are better suited for uncertainty and identifiability analysis, and advocate for studies which make use of such or related techniques that better characterise the parameter space and to report the insights from these extra analyses where possible. Our objective is to test the extent to which PBPK models are identifiable and discuss the consequences for the field. It is well-known that Bayesian techniques are superior for detailed analysis of parameter fitting and identifiability, but the issue is rarely discussed in physiologically based studies and its consequences not widely considered. Likewise, the ‘sloppiness’ (lack of uniqueness of particular parameters or parameter combinations) has been previously discussed by authors such as Gutenkunst et al. [22] and others [23–26]. Gutenkunst et al. concluded that modellers should utilise collective fits of an ensemble of parameter values and to focus upon predictions, not parameter values. However, there are scenarios in which particular parameter values are required or expected, scenarios in which parameter uncertainty is unexpected (multiple parallel organs with “only” one parameter each) or in which parameter uncertainty could be confined to particular parameters, in which cases computationally intensive collective fits of parameter ensembles to all source data are not necessary or more beneficial than simpler techniques. There is a great body of literature on parameter identifiability in biological and pharmacokinetic models (for example, [27–34]). Here, we show that even relatively simple physiologically-based models can yield unidentifiable parameters and discuss simple techniques that can indicate which parameters are affected and potential reasons for it. Though we use three models as an example and focus on cellular kinetics, the mathematics of cell and drug kinetics are often theoretically equivalent in physiologically based models. Uncertainty and identifiability are critical for all physiologically-based models, and more widespread use and reporting of them would greatly enhance confidence in parameter fits from physiologically-based models, potentially improving their reproducibility and utility for translating their parameters into new settings.

## Methods

### Model by Brown et al. (2021)

#### Data

The model was fit to lymphocyte localisation data from the literature, originally published by Smith et al. [35]. The authors introduced radiolabelled lymphocytes into rats and recorded the radioactivity in different organs as a function of time up to 24 h, which is used as a proxy for lymphocyte localisation. The aim of the study was to determine which factors influence lymphocyte migration patterns. They considered the type/source of antigen that the cells are specific for, the places the cells were activated, the lymph nodes they were extracted from and the size of the cells (whether they were effector cells or not). There are several data sets, of which we used one: where lymphocytes were extracted from mesenteric lymph nodes after the donor rat was exposed to environmental antigen.

#### Model

**Fig. 1:**
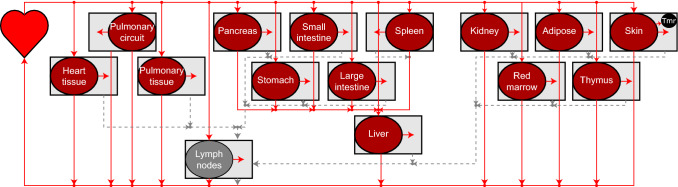
A visual summary of Brown et al’s model of the circulatory system [5]. Solid and dotted lines represent blood and lymph flow, respectively. Cells flow from the heart to each organ, from which a proportion enters the interstitial space. Cells from the interstitium flow via the lymphatics back to the heart. A tumour (“tmr”) can be represented by choosing a tumour bearing organ (the skin in this example) from which proportions of its volume and blood supply are occupied by the tumour (Color figure online)

A set of linear ordinary differential equations (ODEs) were developed to describe the circulatory system and the migration of cells in and out of the blood, as previously described [5]. The equations for organs with no special (circulatory) features are,

1 $$\begin{aligned} \begin{aligned} V_o \frac{\mathrm {d}C_o}{\mathrm {dt}}&= B_o(C_\mathrm {h}-C_o), \\ \tilde{V}_o\frac{\mathrm {d}\tilde{C}_o}{\mathrm {dt}}&= e_o B_o \left( C_o - \mu _o \tilde{C}_o \right) , \end{aligned} \end{aligned}$$

where $V_o$, $C_o$ and $B_o$ are the vascular volume, vascular concentration and blood flow of/to organ *o*, $\tilde{V}_o$ and $\tilde{C}_o$ are the interstitial volume and concentration of/in organ *o*, $e_o$ is the proportion of cells that extravasate out of the organ vasculature rather than returning to circulation, and $e_o \mu _o$ is the rate at which cells leave the interstitial space of the organ via lymphatic vessels. Similar equations can be formed to capture details of the spleen, mesenteric organs, liver, lymph nodes and pulmonary circuit, as presented in Fig. 1. An equation for the heart acts as a mass balance term, receiving blood returning from the vasculature and lymph returning from lymph nodes.

#### Initial estimates for parameters

Fitting this model to data is made more efficient by selecting initial estimates for parameters that are close to the best fit values. These initial values were generated with the following analytical estimates,

2 $$\begin{aligned} \begin{aligned} e^{0}_o&\approx \frac{\tilde{N}_o(t) - \tilde{N}_o(0)}{B_o \int _0^t C_o(t) \mathrm {dt}} \\ \mu ^{0}_o&\approx \frac{C_o}{\tilde{C}_o}, \end{aligned} \end{aligned}$$

where $\tilde{N_o}=\tilde{V_o}\tilde{C_o}$. The estimate for $\mu ^{0}_o$ is to be used as $t\rightarrow \infty$, but the estimate for $e^{0}_o$ must be used as close to $t=0$ as possible, as the right-hand side is time-dependent and neglecting $\mu$ in its definition quickly becomes inaccurate with $t>0$. Current parameter estimates ($e^{n}_o, \mu ^{n}_o$) can be used to gain an improved estimate ($e^{n+1}_o, \mu ^{n+1}_o$) using,

3 $$\begin{aligned} \begin{aligned} e^{n+1}_o&\approx \frac{\tilde{N}_o(t) - \tilde{N}_o(0)}{B_o \int _0^t \left( C_o(t) - \mu ^{n}_o \tilde{C}_o(t) \right) \mathrm {dt}} \\ \mu ^{n+1}_o&\approx \frac{ \int _0^t e^{n}_oB_oC_o(t) \mathrm {dt} - \left( \tilde{N}_o(t) - \tilde{N}_0(0)\right) }{e^{n}_o B_o \int _0^t \tilde{C}_o(t) \mathrm {dt}}, \end{aligned} \end{aligned}$$

where the data or an estimate of the data can be used to provide values for $\tilde{N_o}$, $C_o$ and their integrals. Both of these equations are modified for organs such as the liver or lymph node. Fitting was ultimately performed using curve_fit or basinhopping from the SciPy library in Python [36], and organs were iteratively fit-one-at-a-time. The exact strategy is detailed in Online Appendix Section A.2.

#### Scoring a fit

We made use of the curve_fit or basinhopping algorithms in the SciPy library in Python [36]. curve_fit’s default method is the Levenberg-Marquardt algorithm, which was used in this study. basinhopping uses an algorithm designed by David Wales and Jonathan Doye [45]. It is similar to simulated annealing with a Metropolis criterion; repeated cycles of local minimisation followed by random hops with an acceptance criterion. The local minimisation can use one of several methods, and Sequential Least Squares Programming was used in this study.

The metric for scoring data was a simple sum of square of the differences between model estimates and data over each organ and time point. Throughout this study, we refer to “good” and “bad” fits. Quantitatively, a “good” fit is a low score, which is a small value of the sum of squared differences between model estimates and data. More often, references to a “good” fit in this study are simply how close model estimates and data appear to be, by qualitative inspection.

#### Timescales associated with parameters

By considering the timescale on which vascular and interstitial compartments of the model (Eq. 1) reach equilibrium, we can derive characteristic timescales for each parameter and organ:

4 $$\begin{aligned} \begin{aligned} \tau _{e_o}&= \frac{ V_\mathrm {tot} }{ e_o B'_o } \\ \tau _{\mu _o}&= \frac{ \tilde{V_o} }{ e_o \mu _o B'_o }, \\ \end{aligned} \end{aligned}$$

where $V_\mathrm {tot}$ is the total blood volume and $B'_o$ is the effective blood flow to organs, modified for organs such as the liver or lymph nodes. These expressions can be used to determine whether a given set of parameter values would lead to significant changes in model outputs on the timescale of the data, and thus whether the parameter values are likely to be accurately fit. For example, if the timescale associated with $e_\mathrm {pulmonary\ circuit}$ is shorter than available data, then all entry into the pulmonary circuit localisation would occur before the first data point, and it might be expected that this parameter will not be accurately fit or that it is unidentifiable.

### Models published by Ganusov and Auerbach (2014) and Singh et al. (2020)

We repeated our analyses on the models published by Ganusov and Auerbach [10] and Singh et al. [14]. The methods and results are similar to those for the model by Brown et al. and are recapitulated in the appendices.

### Structural identifiability

A prerequisite for practical identifiability (also known as deterministic identifiability) is structural identifiability. This issue is explained and addressed in Online Appendix A.1. The three models presented in this work are structurally identifiable as long as localisation in each compartment is observed. Previous authors have discussed structural identifiability in PBPK models at length, see [37, 38].

### Generation of synthetic data

Synthetic data was generated for all three models in order to test fitting procedures. A set of parameters were chosen and used to run the model, and the output at several time points was used to define synthetic data. The set of parameters used to generate the data was subsequently used as a ground truth, against which least-squares best fit parameters could be compared. When required, noise was added to the synthetic data by multiplying every data point with Gaussian random variates drawn from a distribution with mean 1 and standard deviation $\sigma$, where $\sigma$ determines the strength of the noise. Typically, $\sigma =0.1$ was chosen. This was chosen to be relatively small, yet sufficiently large to cause parameters to become unidentifiable. Strictly, a truncated Gaussian should be used to prevent measurements becoming negative, but this was not used here, as synthetic data was only generated once, and we explicitly confirmed no negative data was used. With a mean of 1.0 and standard deviation of 0.2, the cumulative distribution function of the Gaussian distribution at zero is $2.9 \times 10^{-7}$.

### Sensitivity analyses

Local sensitivity analyses were used on least-squares best fits of the model to data, to determine which parameters have the strongest influence on each model output, and thus are most likely to be well-fit. Here, local sensitivity is defined as the change in score (sum of squared differences between the data and the estimate) after a 1% change in a given parameter. To analyse the relative impact of parameters on each data curve (organ compartment), the scores are subsequently normalised such that their sum is 1.0 for each data curve. This allows comparison of influential parameters across different organs; a parameter that has the highest relative influence on at least one organ compartment is likely to be better fit than otherwise. Analogous results could be obtained with a more sophisticated global sensitivity analysis over limited parameter ranges. The weakness of assessing uncertainty using local sensitivity analyses is that they cannot estimate uncertainty due to the existence of multiple solutions (minima in parameter space), for which a Bayesian approach or a global sensitivity analysis may be more appropriate. In particular, a parameter that has a large influence on data may still be poorly fit if it is compensating for other parameters that are poorly fit, particularly if it has an influence on multiple organ compartments.

### Bayesian techniques

As a direct comparison to the simple curve-fitting approach and uncertainty analysis described above, we used a Markov Chain Monte Carlo technique to fit models to data. The fit was obtained by assuming that noise is Gaussian and directly proportional to the data, using three Markov chains with random start positions, a burn-in of 1000 samples and repeated cycles of 5000 iterations per chain. Cycles were repeated until a target $\hat{R}$ of 1.1 was reached (where $\hat{R}$ denotes the ratio of the variance between chains to the variance within chains, equal to 1.0 for well-mixed, stationary chains. See [39] for its definition). The analysis was implemented in PINTS [39] with the Haario Bardenet Adaptive Covariance Monte Carlo method. We used the standard deviation of parameter values over the endpoint of the three Markov chains as a measure of parameter uncertainty on our best fit and used density plots of the last cycle (last 5000 iterations) of each Markov chain to visually inspect the nature of the uncertainty or unidentifiability.

## Results

### Fitting a PBPK model to data

**Fig. 2:**
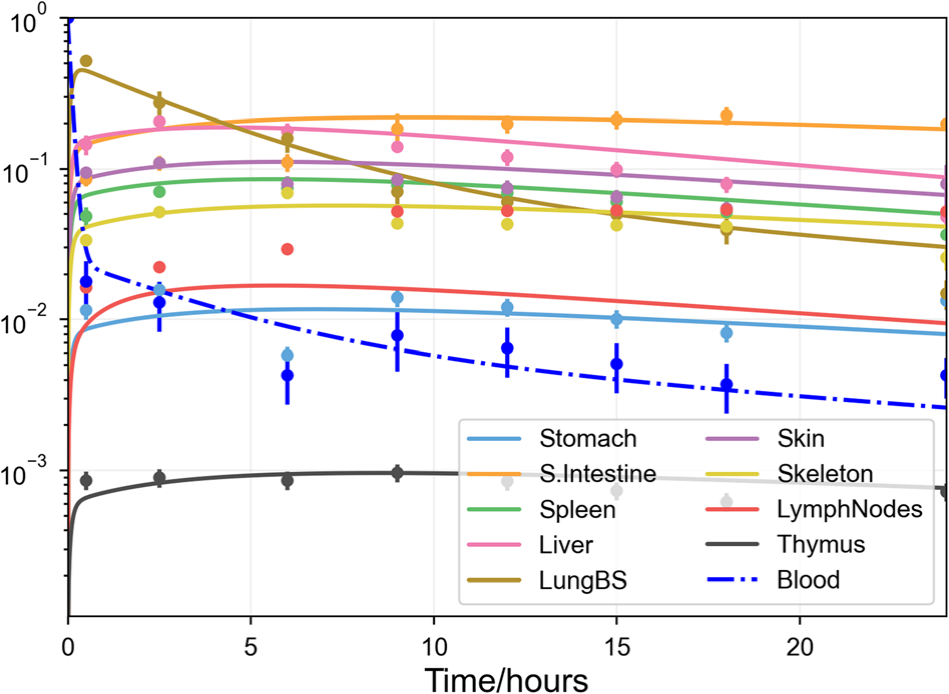
Least-squares best fit of the trafficking ODEs (Eq. 1) to data by Smith and Ford [35], in which lymphoblasts extracted from mesenteric lymph nodes of the rat were radiolabelled and tracked in recipient rats over 24 h with eight time points per organ, assuming that all cells are initially in the blood. Scatter points and bars are respectively the originally published data points and their standard errors. Lines are the model outputs (Color figure online)

Linear systems describing the circulatory system, such as Eq. 1, can be easily fit to data. A least-squares best fit was found for the presented system to data published by Smith and Ford [35], through the procedure described in Online Appendix Section A.2. The results are shown in Fig. 2. The fits to most organs are qualitatively good, matching the time course of the data. The range of parameter values corresponding to good fits is more difficult to evaluate. A measure of uncertainty can be obtained from the local covariance of the fit’s score against parameters, but this does not quantify uncertainty due to valid parameter values elsewhere in parameter space (for example, if the best fit for a model *f*(*x*) occurs at $x=0.5$, but another valid fit can be found at $x=2.0$). To evaluate how accurate we expect these fits to be, we need to make use of synthetic data for which we know the true parameter values.

### Fitting to synthetic data with known parameters

**Fig. 3:**
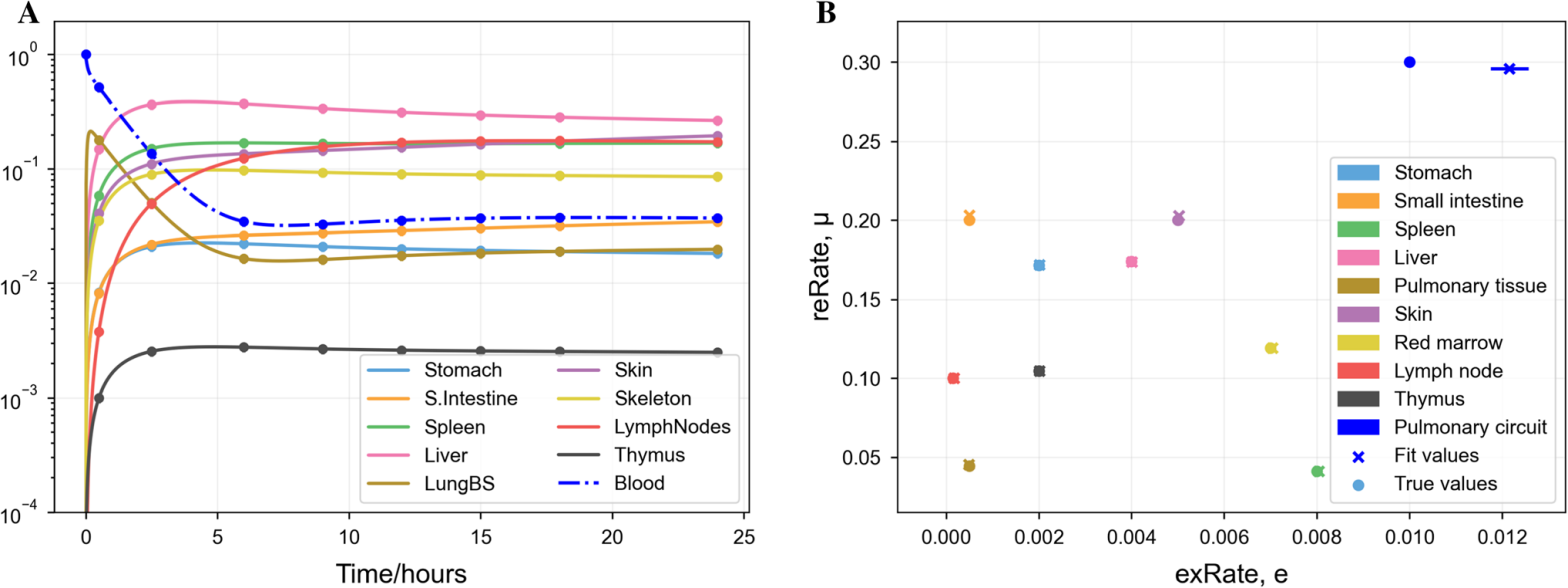
Least-squares best fit of the trafficking ODEs (Eq. 1) to eight synthetic data points plus initial conditions per organ produced by the same model, assuming that initial conditions are that all cells are initially in the blood. **A** Localisation predicted by the model against time. Scatter points (circles) indicate synthetic data. Lines indicate the output of the best fit. **B** A comparison of the “true” values used to generate the synthetic data (crosses) with the best predicted values (circles). The values of µ are plotted on the *y*-axis against the values of *e* on the *x*-axis. Lines show projections of the local covariance of the fit score against parameter estimates (Color figure online)

We generated synthetic data from the model (Eq. 1) using the procedure described in Sect. “Generation of synthetic data”. In order to test the identifiability of the ODE model (whether a given output can be mapped back to a set of parameters), we fitted the model to this synthetic data without using our knowledge of the true parameter values and compared the obtained fits to the true values. These results are displayed in Fig. 3, showing that the data fits exactly (sum of squares between data and estimate of $10^{-4}$), and most fit parameters matched the true values, except for the extravasation probability for the pulmonary circuit ($e_\mathrm {LungPC}$). This may be because the timescale on which this parameter influences localisation in the pulmonary circuit is shorter than the first data point, see Sect. 3.4.1.

### Fitting synthetic data with fewer data points

**Fig. 4:**
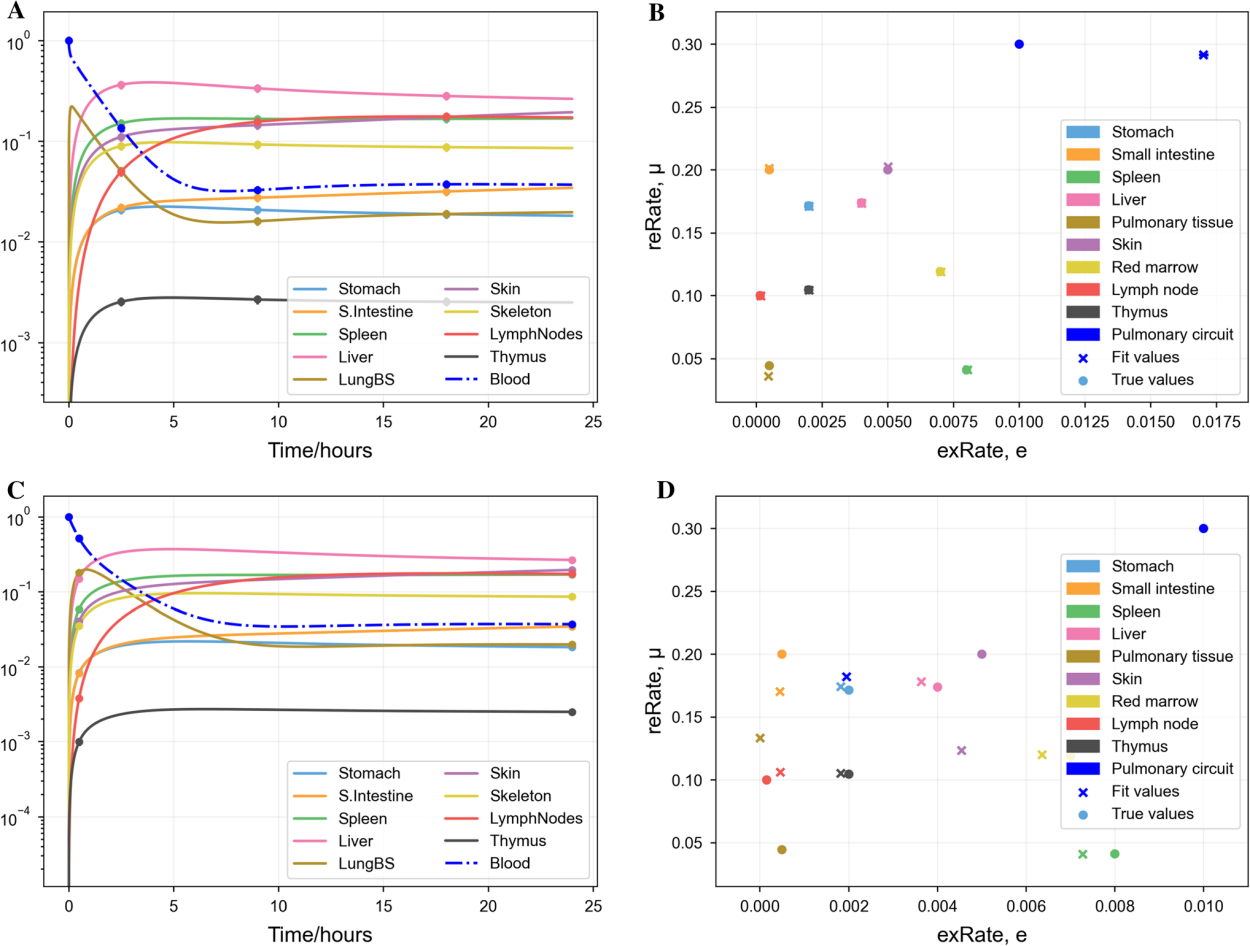
Least-squares best fit of the trafficking ODEs (Eq. 1) to synthetic data produced by the same model, as in Fig. 3, but with fewer synthetic data points. **A**, **B** Fits with 3 data points after the initial. **C**, **D** Fits with 2 data points after the initial, in which parameter estimates begin to get less accurate (Color figure online)

Following the successful fit to a dense synthetic data set, we investigated how few data points were required for the correct parameter values to be recovered from the synthetic data. Results are shown in Fig. 4. With as few as three data points (selected to maximise time between points) per organ after the initial, all fitted parameter values but the extravasation probability in the pulmonary circuit are very similar to their true values. When only two data points after the initial were used, then many parameter values become inaccurate, likely because the transient, short-term behaviour of the model is no longer captured by the data.

### Fitting noisy synthetic data

**Fig. 5:**
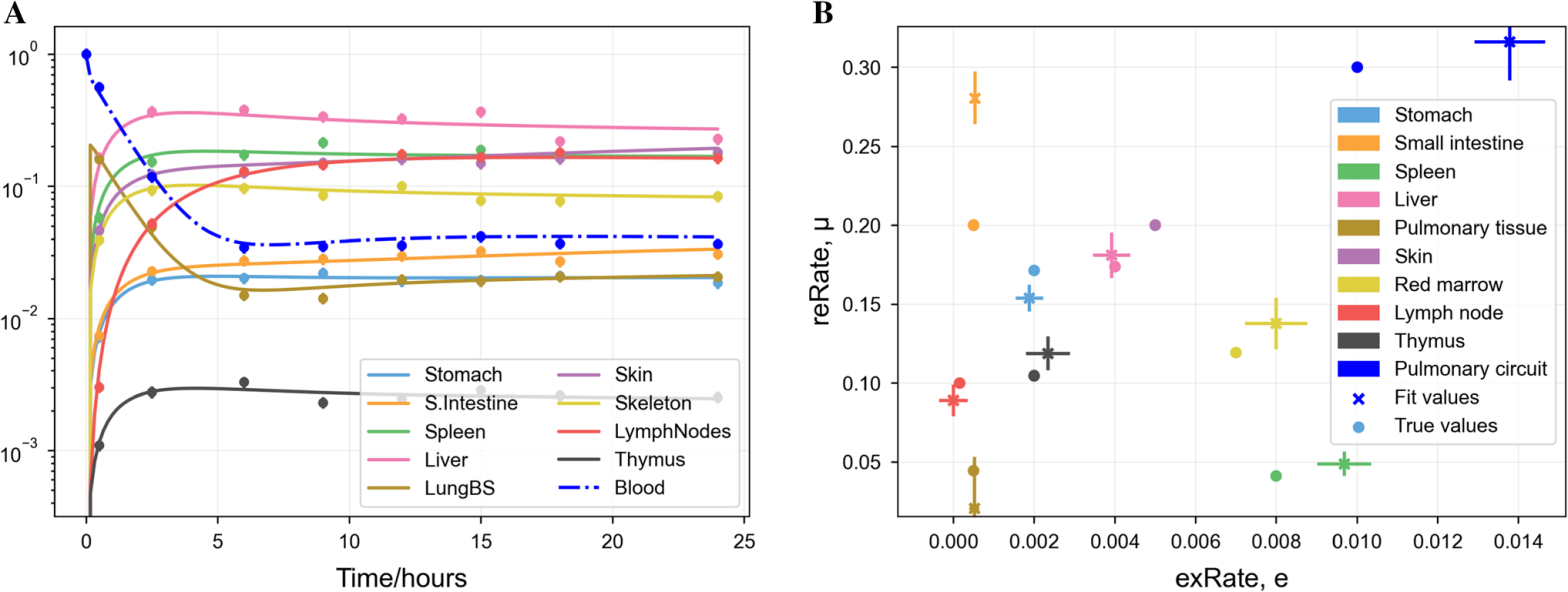
Least-squares best fit of the trafficking ODEs (Eq. 1) to eight synthetic data points plus initial conditions per organ produced by the same model, as in Fig. 3, but with Gaussian noise. Noise was added by multiplying all synthetic data by random Gaussian variates with mean 1.0 and standard deviation 0.1. Fit quality appears qualitatively similar (**A**) but parameter values are fitted much less accurately (**B**) (Color figure online)

Models that are theoretically identifiable, or identifiable with perfect data, may not be identifiable in practice or with noisy data. We multiplied the synthetic data with Gaussian noise of mean 1.0 and standard deviation $\sigma =0.1$ and fitted the trafficking ODE model to the noisy data with the same procedure as before. The results are shown in Fig. 5. The fit to the noisy data is qualitatively good, but many parameter values are now much less accurate. “Standard deviations” corresponding to the main diagonal of the covariance of parameter fits are plotted around the mean of each parameter, but these ranges do not always overlap with the true parameter value. They correspond to how the fit score changes with parameter value locally, which does not directly account for how much of parameter space gives a good fit, nor for the existence of multiple solutions. They thus may be a poor estimate of parameter uncertainty.

**Fig. 6:**
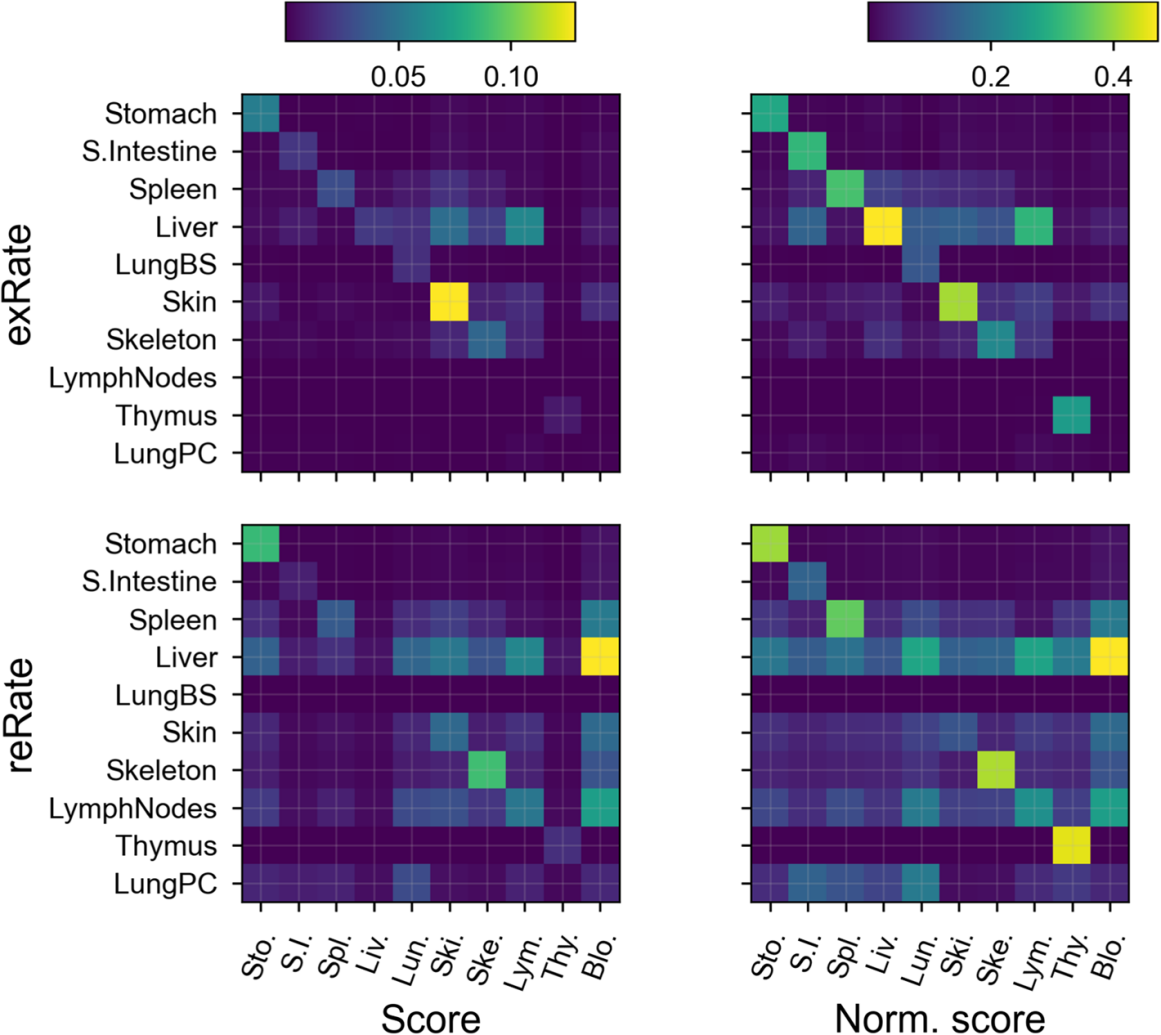
Local sensitivity analysis of the final least-squares best fit to data shown in Fig. 5. Brighter colours indicate higher sensitivity. The top two panels show extravasation rates *e* and the bottom two panels show return fractions µ. Rows correspond to parameter values and columns to different data curves; for example, the last column of the left panels shows that the fit to blood data is most sensitive to the return fraction of cells from the liver. The left two panels show unmodified sensitivity (fractional changes in score). The right two panels are normalised by the sum of changes for each data curve (columns on the figure), so that the most sensitive parameter for any particular data curve can be seen more clearly. Note that column and row names need not match; there is data for blood localisation and explicit model parameters for the pulmonary circuit, but not vice versa (Color figure online)

To further explore uncertainty in parameter fits, we performed a local sensitivity analysis on the model at the best fit (least-squares) parameter values. The results are shown in Fig. 6; the left panel shows the fractional change in the fit score (sum of square of differences between estimate and data) for each organ after each parameter is changed by 1%. The right panel shows the same data normalised by organ, so that the relative importance of each parameter to each organ can be more clearly seen. If the localisation of cells in each organ depended only on parameters for that organ, then the only bright colours would be on the main diagonal of each plot. However, off-diagonal cells are sometimes as bright as the diagonal. For example, the rows for both parameters for the liver have some brightness for many organs, indicating the influence of the liver parameters on all data. The rows for the lung blood supply’s parameters are almost completely dark, indicating little impact on localisation in any organ. This is likely due to the fact that the pulmonary circuit and lung blood supply both correspond to the same data (observed localisation in the lungs), and because the dynamics in the lung occur on a short timescale (see Sect. “Timescales of different parameter values”). These plots give us an indication of which parameters have little impact on the model fit and so may have inaccurate values, but this too is only a local estimate.

#### Timescales of different parameter values

**Fig. 7:**
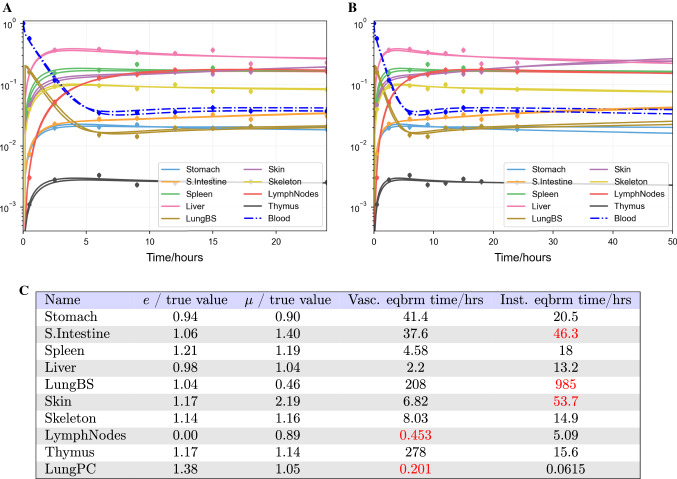
**A** Least-squares best fit of the trafficking ODEs (Eq. 1) to eight synthetic data points plus initial conditions per organ produced by the same model, with data multiplied by Gaussian noise (mean and deviation of 1.0 and 0.1), as in Fig. 5. Plotted on the same axes is the ODE solution resulting from the true parameters. Visually, they are nearly indistinguishable. **B** The same fit extended out to a much longer timescale, on which the true solution and the best fit become marginally distinguishable. **C** Ratios of predicted extravasation rates *e* and return rates $\mu$ to their true values, and the corresponding vascular and interstitial equilibrium timescales calculated from Eq. 4. Highlighted values are earlier than the first data point (30 min) or greater than the final time point (24 h) (Color figure online)

If the least-squares best fit of the model to noisy, synthetic data is plotted on the same axes as the output corresponding to the true parameter values, they may be nearly visually indistinguishable and appear to give equally good fits to the data. However, if the timescale of the output is extended beyond the data, the estimates may begin to diverge. This is shown for the fit from Fig. 5 in Fig. 7. This is a consequence of the timescales on which different organs approach their steady state localisation, in turn due to their parameter values. By calculating the equilibrium timescale for extravasation and return for each organ (Eq. 4), we obtain another indicator of the quality of fit for each parameter. The parameter values and equilibrium timescales for the vascular compartments (determined by *e*) and interstitial compartments (determined by $\mu$), corresponding to the least-squares best fit, are shown in Fig. 7C. The timescales indicate that $e_\mathrm {LymphNodes}$, $e_\mathrm {LungPC}$, $\mu _\mathrm {S.Intestine}$, $\mu _\mathrm {LungBS}$ and $\mu _\mathrm {Skin}$ may be inaccurately plotted. In fact, these are the least accurately fit parameters by their fractional difference from the true values. The local sensitivity analysis (shown in Fig. 6) indicates that the small intestine, liver, lung blood supply, skin, lymph nodes and lung pulmonary circuit may be poorly fit. This list indeed includes the poorly fit parameters, but also parameters which were better fit. This analysis suggests that the timescale on which dynamics occur in each compartment, and the dependence of parameter values on these, can strongly influence the identifiability of individual parameters. In particular, if dynamics occur on a timescale much faster than the first data point, or much slower than the final data point, the corresponding parameters may not be identifiable.

#### Fitting synthetic data with data unrecorded in some organs

**Fig. 8:**
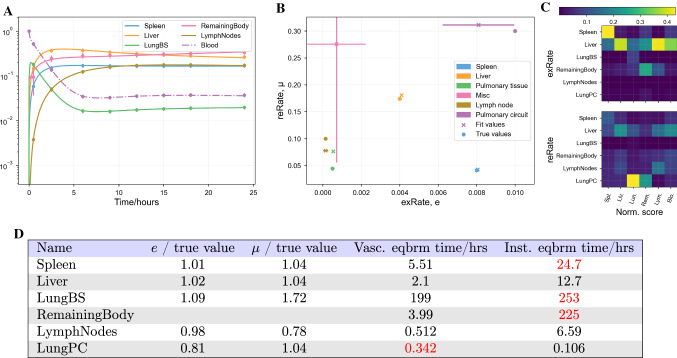
**A** Least-squares best fits of the trafficking ODEs (Eq. 1) to eight synthetic data points plus initial conditions per organ *without* noise as in Figure 3, but with all organs not listed in the table removed from the data after production. **B** Least-squares best fit parameter values versus “true” values. Many parameters have very inaccurate values. **C** Local sensitivity analysis for these parameter values, as described in Figure 6. **D** Timescales associated with fit parameters, as described in Fig. 7C (Color figure online)

Data of the localisation of cells in an organism is typically measured only in a subset of organs, rather than in every organ. We investigated whether fitting a single pair of parameters to organs for which localisation was not recorded strongly impacts parameter estimates for the organs under investigation. A fit to such synthetic data *without* noise is shown in Fig. 8. The fits look perfect, but several parameter values are far off from their true values, and a local sensitivity analysis shows that the liver dominates local changes in the score for most organs. A timescale analysis shows that some of the worst-fit parameters are those whose equilibrium timescales are outside of the range of data, as before, but in this instance the lymph nodes parameters are also poorly fit. These results suggest that censoring some organs from the data, in which localisation is not insignificant, may cause compensatory changes in estimated parameter values for measured organs.

#### Fitting synthetic data from a system with fewer organs

**Fig. 9:**
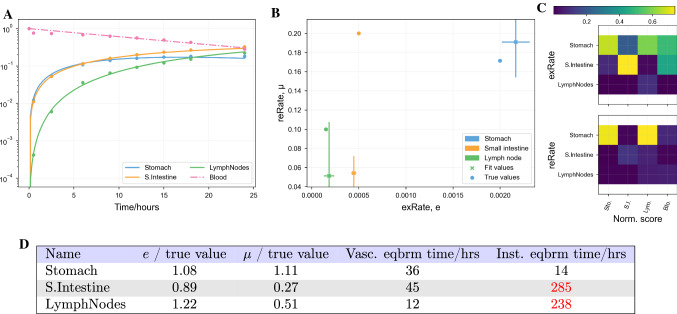
Least-squares best fits of the trafficking ODEs (Eq. 1) to eight noisy synthetic data points plus initial conditions per organ. Noise was generated by multiplying data by a Gaussian of mean 1.0 and standard deviation 0.1. Rather than removing existing organs from data, this plot corresponds to synthetic data acquired from an ODE system in which only the three displayed organs exist. Parameter values still do not always fit. Explanations for each figure panel areas in Fig. 8 (Color figure online)

We have also tested a smaller system of ODEs to determine whether parameters are easier to determine with fewer organs. We created trafficking ODEs akin to Eq. 1 in which the only organs with blood flow are the stomach, small intestine and lymph nodes, then created synthetic data. When synthetic noise is added to the data, however, parameters are still not identifiable; parameters that are very different to the true values produce fits that are as good as the true fit by eye, as shown in Figure 9. A local sensitivity analysis indicates that stomach return rate dominates other parameters. Despite this, even the stomach parameters are not well-fit. A timescale analysis shows that, as before, the timescale for equilibrium of the other two compartments lies far outside the timescale of the data. This suggests that a close relationship between organ compartments prevents identification of physiological parameters, even for small systems, if data on an appropriate timescale is not available.

Open loop modelling, where the concentration in the blood compartment (arterial supply) is used as a forcing function for an organ compartment, can be used to focus modelling on a single organ with one or two parameters. The analysis of the ODE system with fewer organs suggests that, because organ compartments are not completely independent, the parameters obtained from open loop modelling may not be equal to the parameters for the same compartment in the full organ system.

#### Fitting synthetic data with some parameters fixed

**Fig. 10:**
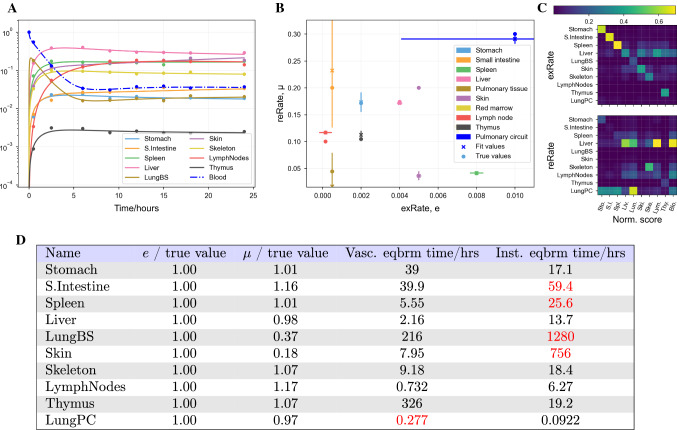
Least-squares best fits of the trafficking ODEs (Eq. 1) to eight noisy synthetic data points plus initial conditions per organ as in Fig. 5, but with *e* fixed to true values. Noise was generated by multiplying data by a Gaussian of mean 1.0 and standard deviation 0.1. Fits to $\mu$ are still not perfectly accurate. Explanations for each figure panel are as in Fig. 8 (Color figure online)

We tested whether parameters are identifiable if other parameters are held fixed. We fixed all values of *e* to the true values used to produce synthetic data, and fitted the remaining values $\mu$ after the data had been multiplied by Gaussian noise (mean and deviation of 1.0 and 0.1), as before. Results are shown in Fig. 10. Though fits to the synthetic data look good by eye, the values of $\mu$ are far from their true values for most organs, and a local sensitivity analysis indicates that the score is most sensitive to changes in *e*, not $\mu$. A timescale analysis shows that the poorly-fit parameters once again have equilibrium timescales far outside the range of the data. This suggests that, even with known or assumed values of parameters, remaining physiological parameters may still not be accurately fit.

### Bayesian Computation

**Fig. 11:**
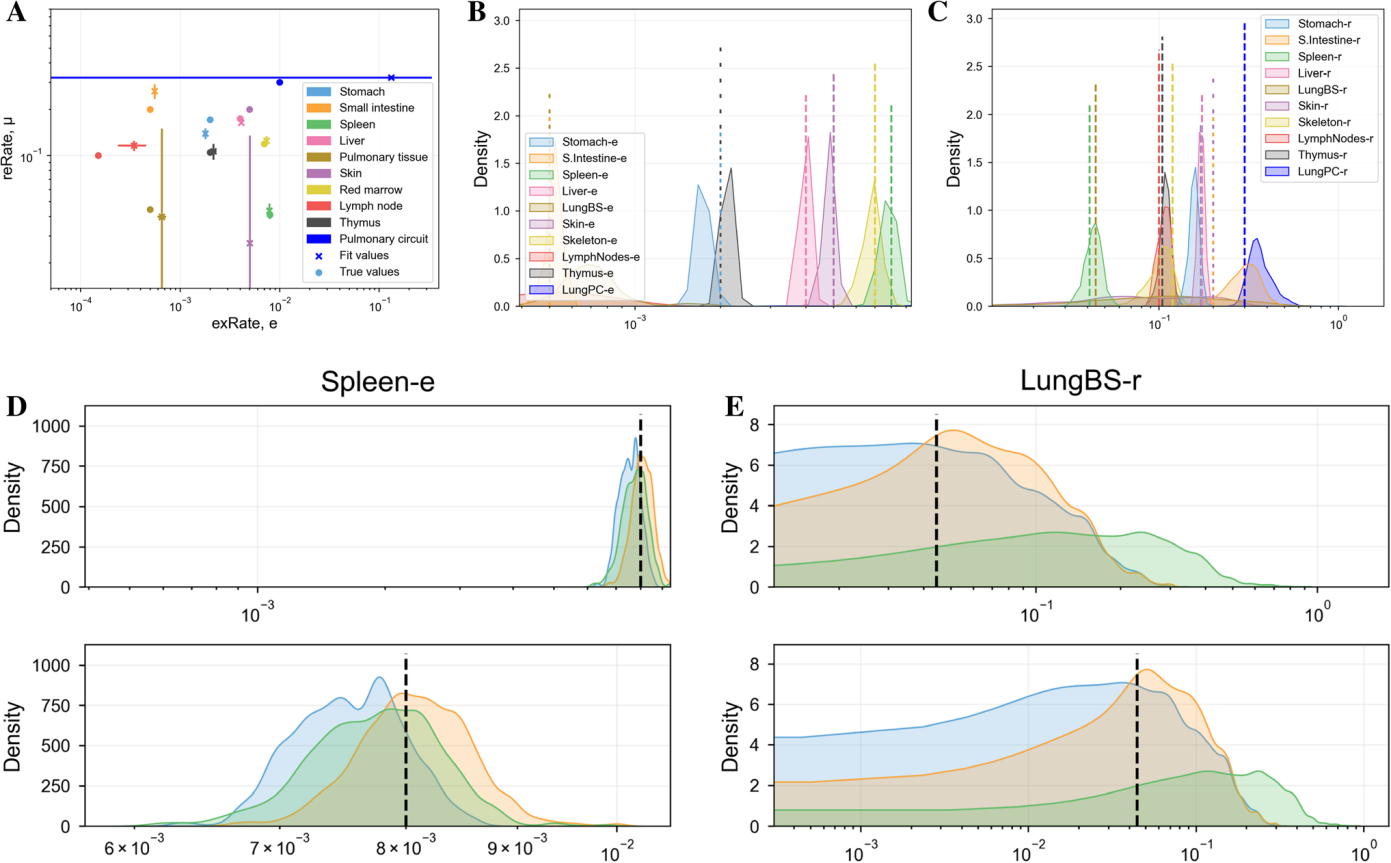
The distribution of parameters produced by fitting the ODE system in Eq. 1 to eight noisy synthetic data points plus initial conditions per organ, using adaptive covariance Monte Carlo [39]. Noise was generated by multiplying data by a Gaussian of mean 1.0 and standard deviation 0.1. **A** Best fit parameter values (crosses x) and the true values (circles o) for each fit. Standard deviations (vertical lines) are calculated across the final value of the three Markov chains. **B**, **C** Density plot of values of *e* (panel **B**) and $\mu$ (panel **C**) across the last 5000 samples of every Markov chain. Dashed lines indicate true values of parameters. **D**, **E** Density plots for the values of $e_\mathrm {spleen}$ and $\mu _\mathrm {LungBS}$ in the last 5000 samples of each Markov chain in turn. Dashed lines indicate true values of parameters. The top and bottom half of each panel shows the same density plot on different axes ranges; the top half shares axes limits with **B** and **C**. The spleen density has a much narrower peak than the lung blood supply density (Color figure online)

To demonstrate how a Bayesian approach may better identify poorly fit, non-identifiable or sub-optimally fit parameters, we used a Markov Chain Monte Carlo technique [39] to fit synthetic data to the trafficking ODEs defined in Eq. 1. As described in Sect. “Bayesian techniques”, we fit the model to data by repeating cycles of 5000 iterations for each of three Markov chains until $\hat{R}$ fell below 1.1 (see Sect. “Bayesian techniques” and [39] for its definition), starting from Gaussian priors around initial estimates. For Eq. 1, this required a total of 440,000 samples per chain. Results are shown in Fig. 11.

Panel A shows true parameter values (used to generate the synthetic data) against best fit parameters and their standard deviations, calculated from the final parameter values in the three Markov chains. Unlike in previous plots, these standard deviations typically do overlap the true values. Panels B and C show density plots of parameter values sampled in the last cycle of the MCMC fit, i.e. the last 5000 iterations of each Markov chain or 15,000 parameter sets in total, for extravasation rates (panel B) and return rates (panel C). Some parameter densities are highly peaked (indicating a good fit) and others are not (indicating an unidentifiable or poorly fit parameter). Panels D and E show the density plots from the last 5000 samples of each chain separately, for the spleen extravasation rate (panel D) and lung blood supply return rate (panel E), showing how densities for well- and poorly-fit parameters differ.

### Other models

To discount the possibility that the lack of identifiability is a feature only of the model described in Eq. 1, we repeated the above analyses on the models published by Ganusov and Auerbach [10], and by Singh et al. [14]. These results are shown in the appendices. As with the Brown et al. model, both models produce close fits to synthetic data with parameter values different to those used to produce the synthetic data. That is, both models have practically unidentifiable parameters.

## Discussion

Physiologically-based pharmacokinetic (PBPK) models are used extensively in academia and in industry to model the distribution of drugs and their dynamical effects on the body. Increasingly, such models are used for cellular kinetics, to study the distributions of cells such as CAR T-cells. Kinetic models are often linear and are relatively simple to fit to data. However, the circulatory system is highly connected, resulting in parameters for one organ compartment having a non-negligible impact upon the data fits to other organs. Different sets of parameter values can therefore produce qualitatively similar fits. When parameter values are unimportant compared to the fit itself, this is not a problem, but where some biological meaning is sought from the parameter values, this may lead to incorrect interpretations, and when fits to different data sets, patient populations or timescales are to be compared, additional analysis may be required to establish confidence in predictions. We have demonstrated this problem with three published physiologically-based models of cellular kinetics, each of which give visually good fits to data and were fit for their purposes. For each case, we showed how synthetic data made from known parameter values can be perturbed with random multiplicative Gaussian noise, and how this noise prevents accurate recovery of the original parameters (Sect. “Fitting noisy synthetic data”, Online Appendix Sections B.1, B.2). The fundamental reason for this is that the sum of the squared differences between the noisy data and the true data becomes similar to the difference between non-optimum solutions and the data, in turn due to these non-optimum solutions being too qualitatively similar to the true solution, despite the different parameter values. A plot of fit score as a function of parameter value would reveal regions of parameter space that have the same or a lower score than the true solution, i.e. that parameters are practically unidentifiable. This problem is easy to visualise for models with one to three parameters, as shown in [40]. Another issue with fitting systems with many parameters is that similar solutions can have parameter values that are very far apart in parameter space, separated by large potential barriers (poor solutions) that make crossing from a non-optimum solution to a better solution difficult. This is particularly relevant for physiologically based models, which require many parameters: typically, one or two per organ in a system of ten or more organs of interest. Many parameter fitting techniques cannot adequately handle the high dimensionality of such systems. A crude Latin Hypercube with *n* evenly spaced values in each of *p* parameters requires $n^p$ evaluations of the model. If a given physiologically-based model contains 10 organs with unknown parameters, and two parameters per organ, then just three values per parameter would require $3^{20} = 3.5 \times 10^9$ evaluations, which would require 110 years if each evaluation took one second.

The most common tool used to fit PBPK models to data are simple curve-fitting techniques (9 of 13 from a selection of recent, highly cited studies [6–11, 14, 16–21]), which are prone to returning non-optimum solutions in systems with many parameters. For the three models presented in this study, localisation within an organ is primarily sensitive to that organ’s own parameters (by global sensitivity analysis; data not shown), so it is possible to fit parameters to data one-organ-at-a-time (among other strategies; see Onine Appendix Section A.2). Whilst this strategy leads to a good fit in most cases, parameters are even more vulnerable than normal curve-fitting approaches to becoming trapped in local minima, as a better solution might require a change to multiple organs’ parameters. If changing the parameters for any single organ would result in a worse solution, then this strategy will not be able to reach the better solution. This can potentially be remedied by strategies such as scaling all extravasation or return rates by the same factor after the organ-specific fit cycle or fitting the *n* most altered parameters to the data as a group, but this problem cannot be completely overcome as long as parameters are fit in isolation to other parameters.

These problems aside, in the absence of noise, the true parameter values could be recovered with all three models. However, multiplying by a Gaussian of mean 1.0 and standard deviation of 0.1 results in least-squares best fits that look qualitatively similar to the data and the true solution, but with different parameter values. As already stated, this is because the noise is a similar size to the difference between optimum and non-optimum solutions. Of the three models presented, the one published by Singh et al. [14] was slightly more robust to noise. Parameters could be recovered until the standard deviation of the noise Gaussian reached 0.2, because there were fewer organs and only one parameter per organ, making non-optimum solutions further from the optimum than the other models. However, this was with the caveat that several parameters had to be given fixed values. The absence of blood data in their publication leads to degeneracy in the transmigration rates, as any transmigration rates that lead to entry into organ compartments on a fast enough timescale yield the same interstitial localisation profiles at later times, as long as the relative transmigration rates remain the same. Without blood data to fit the magnitude of transmigration rates, data fitting is more complicated and can result in local solutions that are significantly worse than the least-squares best fit, but separated from it by high potential barriers. To remedy this, we fixed the same parameter values that Singh et al. fixed to literature values, originally due to an absence of data for those organs. We then set the mean value of initial estimates of parameter values to be the same as the mean of the fixed values. The constrained parameter values may result in initial parameter estimates being closer to the region of parameter space that contains the optimum fit than otherwise, and might have made model fitting more robust to noise.

Since non-optimum solutions are selected due to their score (sum of square of differences between the estimate and the data) being similar to the size of the noise, a system with fewer organs might be fit more robustly and the parameters might be more identifiable. Results in this manuscript show that this is only true to an extent. When a system that only has three organs is used to create synthetic data, multiplicative Gaussian noise still resulted in incorrectly fit parameters (Fig. 9). Synthetic data produced from the model implemented by Singh et al. was much more robust to Gaussian noise, but this was due not only to the smaller number of organs, but that there was only one parameter per organ, and because the fit was seeded with several fixed parameter values, which influence the possible values of other fit parameters.

A similar concern is whether assumptions made about parameters for which organ data is missing have large impacts on other parameter values. We censored some of the organs from the synthetic data (i.e. some of the cell localisation was unaccounted for) before repeating the data fitting process. When a single pair of parameters were assigned to ‘other’ (non-data) organs, least-squares best fit parameter values for several organs deviated from correct values to compensate, even in the absence of noise (Fig. 8). This indicates that when significant amounts of localisation are unaccounted for in a modelling study, parameter values obtained from the data may not be trustworthy.

Since parameters only become non-identifiable in the presence of noisy data for these models, it could be the case that having a greater number of data points would improve parameter identifiability. As before, this is only true to an extent. More data points means that a curve that passes close to all of them is more likely to follow the trajectory of the true solution. In addition, the existence or absence of data on the timescales of equilibrium for various organ compartments allows or prevents accurate fitting of the corresponding parameters. However, the fundamental reason for non-identifiability of parameters is not the noise, but that qualitatively similar solutions can be obtained with very different parameter values. Real-world data is unlikely to produce data curves that exactly correspond to model outputs (i.e. it is unlikely that a given PBPK model is perfect), and so the difference between the data and a given model output will in general be much greater than the small differences between these similar solutions. In other words, identifiability of parameters will typically be much worse for fits to real data than to synthetic data as presented here. Thus, even with very rich data, parameters are likely to continue to have multiple possible fits.

The obvious remedy to this issue is to calculate uncertainty in all parameter estimates. For non-Bayesian, simple curve-fitting techniques, one can obtain ‘uncertainties’ from the main diagonal of the local covariance matrix of the fit score to changes in parameters. Here, the fit score is the sum of squared differences between the data and the solution. As indicated by examples in this study, the ‘uncertainty’ obtained from the local covariance represents the local gradient of the score to each parameter, *not* the actual parameter uncertainty. It does not necessarily overlap the true parameter value, because the true parameter value may be in a very different region of parameter space and the score might be robust to small, local changes in the estimated parameter value. A local sensitivity analysis can give a hint to which parameters have little impact upon the fit and so may not have their true values, but this too is a local estimate with the same weaknesses as use of the local covariance.

A more useful indicator of whether a parameter might be poorly fit to data is the timescale to equilibrium in some compartment associated with it. If such a timescale is much shorter or much longer than that of available data, then changes in this parameter value will have little impact on the fit to the data. This analysis is still not infallible, as some parameters that have influence on multiple data curves might take an ‘incorrect’ value to better fit all data curves, or to compensate for other incorrect parameter values. The importance of equilibrium timescales is best illustrated by extending fits to data out to longer timescales. Curves with different parameter values that look similar on one timescale may become distinct on a different timescale, as shown in Fig. 7. Data on this timescale would be better able to differentiate between the two parameter values. Consideration of the timescale or timing of biological mechanisms is relevant to optimal design of experimental measurements. Depending on the experiment and the model, parameters may be more identifiable and/or the model may be more informative if the density of measurements match the timescales of the dynamics driven by the parameters of interest. In some cases where physiologically-based pharmacokinetic models are used, all that is required is a good fit to data, not perfect parameter values. However, these models are often extrapolated to different timescales or different populations. This example makes clear that extrapolation to other timescales would produce different answers with different sets of parameter values that otherwise gave very similar fits to data. If the new timescale is very short, it might also be the case that ODEs become inappropriate as cells redistribute among organs, because ODEs assume an exponential distribution in cell residence times within organs. Similarly, extrapolation to another population (such as a paediatric population) may require scaling of particular parameter values, but if these are not fit with a unique value, such scaling might not be meaningful, or appropriate. In other studies of physiologically-based pharmacokinetic models, the models are intended to be exploratory simulations that draw upon several in vivo and in vitro sources of data, and are not fitted to data. In this case, parameter identifiability is not as simple to verify without generating synthetic data, but would cause many of the same problems: several sets of parameter values may yield the same output. If the qualitative behaviour of the model is the only important output, then identifiability is not a concern, but if parameter values have important biological meanings, are to be scaled to other populations or timescales, or are reported and subsequently used by others, then even in this case, it is important that parameter uncertainty and identifiability are explored.

An even better estimate of the identifiability of parameters in a given fit can be obtained from a profile likelihood, which quantifies the marginal probability of generating data of interest given the chosen model and variable parameter values. This is more computationally expensive than local sensitivity or timescale analyses, but gives a true estimate of uncertainty, as opposed to the local covariance of the fit score against parameters. It was not shown here, in lieu of Markov Chain Monte Carlo, which yields similar uncertainty estimates and can additionally be used to *fit* data from scratch. Profile likelihoods, through the likelihood function, are closely related to a class of techniques that is one of the best suited for exploring parameter spaces with multiple local minima: Bayesian inference. Bayesian inference algorithms include Approximate Bayesian Computation (ABC), Markov Chain Monte Carlo or Stochastic Approximation Expectation Maximisation (SAEM), the latter of which is utilised by commercial solutions such as Monolix and Adapt V. However, though more efficient than other techniques, Bayesian techniques still become inefficient as the dimensionality of the parameter space becomes large, and selection of priors can have a large impact on parameter fits. For example, Gaussian priors far away from the global optimum may result in the global optimum not being found, and wide uniform priors may make fits converge very slowly. Efficient handling of the ‘curse of dimensionality’ is an active field of research, both in Bayesian and frequentist contexts [41–44]. We fitted the trafficking model presented in Eq. 1 using Adaptive Covariance Monte Carlo implemented in PINTS [39], as detailed in Sect. “Bayesian Computation”. We iterated three Markov chains with random initial starting points until the variation between the chains became similar to the variation within the chains. The standard deviations of our best fit parameters were then calculated across the final values of the three chains. As these represent actual variation in possible solutions, we would expect this measure to more reliably capture uncertainty in parameters than local covariance of the fit score, and the error bars plotted in Fig. 11B do indeed overlap the true values. Density plots obtained from a Bayesian technique are also useful in analysing fits, as the existence of multiple minima or non-identifiable parameters can be quickly verified. Figure 11D, E shows density plots of the last 5000 iterations of each Markov chain for two parameters. The extravasation rate into the spleen, panel D, has a well-peaked distribution and we would expect a good fit for this parameter. The return rate from the lung, panel E, has a heavy tail towards the right-hand side of the plot, and a plot of all iterations from each chain (not shown) indicates the presence of multiple minima. Supplementary Figure S4E shows an additional case, where density is uniform everywhere, for a parameter that is completely unidentifiable. Though we made similar inferences from the fits and local sensitivity analyses made in Sect. “Fitting noisy synthetic data”, they were made with less precision and confidence than from Bayesian results. We could not be sure if the simpler fit obtained was a global minimum, whereas the use of multiple Markov chains allows simple verification of the number of minima (if any) present.

Though it is not a surprising result that Bayesian techniques are better at quantifying parameter uncertainty, we have demonstrated that such techniques are useful, if not required, in the analysis of PBPK models. However, much of the literature of physiologically based models does not make full usage of Bayesian techniques, likely due either to large numbers of model parameters, or because authors need close fits to data but not accurate parameter values. This may, however, influence conclusions or analysis from other models. For example, mathematical modellers interested in the same biological system may search the literature for useful data or parameters and make use of the values without understanding the uncertainty or identifiability in the original model, or the original work might be extended or applied to a new context that is parameter-dependent. Other authors, such as Gutenkunst et al. [22–26], have suggested alternative approaches such as finding the ensemble of all parameters sets that fit the data, and presenting the mean and confidence intervals of results from these parameter sets as the model output, as in a virtual patient/subject population. As argued in our introduction, this may not always coincide with the study aims, an estimate of the uncertainty on particular parameters may sometimes be desirable (rather than results), and such an approach may nonetheless require Bayesian techniques, which can be used to report on parameter identifiability and uncertainty as we have suggested.

## Conclusions

Physiologically-based pharmacokinetic models are used extensively in academia and industry to fit concentration profiles of drugs or cellular therapeutics to data. The fits obtained from such models can be used to calculate quantities such as the area under the curve, in turn used for quantities such as the overall exposure to a drug. Such quantities are independent of the parameter values used to obtain the fit. However, other useful quantities depend directly on parameter values, such as half-lives in particular compartments, or the scaling of model behaviour to other populations. In this study, we have explored whether parameters used to generate synthetic data are practically identifiable (also known as deterministically identifiable) and can be recovered when noise is added to the data. We have found that physiologically-based systems are highly interlinked, and often yield disparate sets of parameters that produce very similar fits to the data. We verified this in three different ODE systems. When fits are imperfect or data is noisy, these fits cannot be visually distinguished, even with large difference in parameter values. Sets of parameter values may be separated by large potential barriers (i.e. regions of parameter space with a poor fit to data), and physiologically-based models usually have large numbers of parameters due to the number of organs involved. These problems make traversal of parameter space inefficient and difficult and frustrates quantitation of the uncertainty on parameter values. Acquisition of data at different timescales may allow one to differentiate between similar fits, if parameters have impacts on the model that are not already on the timescale of existing data, but this is not always feasible. We showed that global sampling of a model’s parameter space with techniques such as Markov Chain Monte Carlo may be necessary to give reliable estimates of parameter uncertainty and identifiability. In particular, parameter estimates obtained from physiologically-based models may often not be the global optimum or may be one of several similarly well-fitting optima, even when the model fits exceptionally well to data. Such parameters values should be treated with caution, particularly under extrapolation to applications in different populations or model systems. To remedy this issue, uncertainty and identifiability of parameters should be quantified more carefully across the PBPK literature, and efficient sampling techniques, such as Bayesian methods, should be utilised to explore parameter spaces and return regions of parameter space that fit data, rather than point-estimates. More widespread reporting of parameter uncertainty analysis and regions of parameter space that fit data would have a positive impact on model interpretation, translation and confidence in results, aiding development of future models and their role in supporting clinical decision-making.

## Supplementary Information

Below is the link to the electronic supplementary material.

Electronic supplementary material 1 (PDF 2074 kb)
