# Supplementary material for: Analysis of cellular kinetic models suggest that physiologically based model parameters may be inherently, practically unidentifiable
Source: J Pharmacokinet Pharmacodyn. 2022 Aug 6;49(5):539–56. doi: 10.1007/s10928-022-09819-7 (PMC9508223; doi:10.1007/s10928-022-09819-7)
Supplement: Supplementary file 1 — Electronic supplementary material 1 (PDF 2074 kb) [file 10928_2022_9819_MOESM1_ESM.pdf]

Supplementary material: Analysis of cellular kinetic models suggest that physiologically based model parameters may be inherently, practically unidentifiable

Liam V Brown*[1,2], Mark C Coles[2], Mark McConnell[3,4], Alexander V Ratushny[3], and Eamonn A Gaffney[1]

[1]Wolfson Centre for Mathematical Biology, Mathematical Institute, University of Oxford, UK
[2]Kennedy Institute of Rheumatology, University of Oxford, UK
[3]Bristol Myers Squibb, Seattle, WA, USA
[4]Currently Chinook Therapeutics, Seattle, WA, USA

August 4, 2022

A Appendix: Supplementary Methods

A.1 Structural identifiability

In the main text, we analyse and discuss the practical identifiability of three different PBPK models, *i.e.* the extent to which parameters values can be determined for given data. If the models are not structurally identifiable, *i.e.* the parameters can *never* be uniquely determined from perfect data. For example, if parameters are not independent, then they cannot be uniquely determined and models cannot be practically identifiable either. The three models discussed here can all be written down as linear algebraic models of the form $\frac{\mathrm{d}\boldsymbol{N}}{\mathrm{dt}} = \boldsymbol{A}\boldsymbol{N}$, where $\boldsymbol{A}$ is a matrix encoding ordinary differential equations (ODEs) and $\boldsymbol{N}$ is a vector of the localisation of cells in each model compartment. Following the arguments from Castro and de Boer, the derivatives are structurally identifiable if they are linearly independent, which requires that $\boldsymbol{A}$ is full-rank, *i.e.* that each output and each input of the model is linearly independent. Another way to state this is that the weighted sum of each derivative,

$$\sum_{i=0}^{n} a_i \frac{\mathrm{d}N_i}{\mathrm{dt}}$$

is zero if and only if $a_1 = \ldots a_n = 0$ [1]. The biological parameters that make up the elements of $\boldsymbol{A}$ are in turn structurally identifiable if they are linearly independent from each other (do not only appear in a product or sum with other parameters, for example). Using the method published by Castro and de Boer [1] for the three ODE systems considered here, after removing the mass balancing (heart) equations that make the functions linearly codependent, one finds that all parameters and variables are structurally identifiable if there is an observation in each organ compartment and the blood.

In practice, ensuring linear independence is not always strictly necessary for structural identifiability. Consider a system with two compartments: the subcutaneous layer of the skin into which a drug is injected, and the draining lymph. One could write a simple ODE for the loss of drug from the skin: $\frac{\mathrm{d}N_{\mathrm{SC}}}{\mathrm{dt}} = -dN_{\mathrm{SC}}$, where N_{SC} is the subcutaneous drug concentration and d is the drainage rate. The corresponding equation in the draining lymph, ignoring later loss to the lymph nodes or blood, is $\frac{\mathrm{d}N_{\mathrm{blood}}}{\mathrm{dt}} = dN_{\mathrm{SC}}$. These equations are clearly not linearly independent, but both of the dependent variables can be determined and d is identifiable, *if* we have observations

*Email: brown at maths dot ox dot-ac dot uk

of either dependent variable. Likewise, when fitting to data to the three ODE systems considered here, removal of the heart equation from these models is not required for structural or practical identifiability.

Another more explicit argument can be made if the matrix of system ODEs $\boldsymbol{A}$ is diagonalisable, non-autonomous and non-singular. In this case, the matrix's eigenvalues do not depend on one another, $\boldsymbol{A}$ can be expressed in terms of the matrix of eigenvalues and change of basis matrix $\boldsymbol{A} = \boldsymbol{V}\boldsymbol{\Lambda}\boldsymbol{V}^{-1}$, and the solution to the model can be given by

$$\boldsymbol{N}(t) = \exp\left(\boldsymbol{A}\left(t - t_0\right)\right)\boldsymbol{N}(t_0) = \exp\left(\boldsymbol{V}\boldsymbol{\Lambda}\boldsymbol{V}^{-1}\left(t - t_0\right)\right)\boldsymbol{N}(t_0).$$

Since the eigenvalues of $\boldsymbol{A}$ are do not depend on one another, they can be uniquely expressed in terms of values of $\boldsymbol{N}$, and each eigenvalue λ_i can be identified as long as there is a measurement for the corresponding N_i and initial conditions $N_i(t_0)$ are known. The eigenvalues are necessarily equations of the model parameters, and so the model parameters can in turn be uniquely expressed in terms of identifiable eigenvalues if there are as many parameters as eigenvalues. The ODE system in equation 1 has $2n + 2$ equations, where n is the number of organs: 2 for each organ, plus the heart and tumour-invading lymphocytes. For each organ o, there are two unknown parameters e_o and μ_o, and e_{tmr} for the tumour-invading lymphocytes. The equation describing localisation in the heart is a mass-balancing equation, such that the sum of all system derivatives is zero, so the heart equation is NOT linearly independent of the others. However, the heart compartment can be trivially eliminated from the matrix of equations, leaving $2n + 1$ equations, eigenvalues and parameters. Thus, every parameter is structurally identifiable. A similar argument holds for the other two models considered here.

A.2 Fitting models to data

The system of equations defined by Equation 1 can be very large, depending on the number of organs. With ten organs to consider, there are twenty unknown parameters: e_o and μ_o for each organ o. Simple curve-fitting techniques begin to struggle when the number of parameters gets this large, and several common routines (such as *curvefit* or *basinhopping* in python) will simply return the initial parameter guess. A sensitivity analysis of the localisation of cells in each organ against parameters indicates that organs are more sensitive to their own parameters than others (data not shown), suggesting that organs can be fitted one- or few-at-a-time. This procedure is outlined in Algorithm 1.

A.3 Full system of model equations

The full system of equations describing the exchange of material between the heart and organs, corresponding to Equation 1, is as follows. For an organ o, C_o is the concentration of the cells of interest in the organ vasculature o, $\tilde{C}_o$ is the concentration in the interstitial space, B_o is the blood flow to the organ, V_o is the volume of the vasculature, $\tilde{V}_o$ is the total organ volume, e_oB_o is the rate of extravasation into the organ interstitial space from the vasculature, and $e_o\mu_oB_o$ is the rate of return of cells from the interstitial space via lymph. Some organs are treated specially, and they are abbreviated to "LN": lymph nodes, "h": heart, "PC": pulmonary circuit and "TBO": tumour bearing organ. The equations are given without further comment here; their explanation and interpretation has been previously published [2].

Organ vasculature:

$$V_o \frac{\mathrm{d}C_o}{\mathrm{dt}} = B_o(C_\mathrm{h} - C_o), \tag{1}$$

Organ interstitial space:

$$\tilde{V}_o \frac{\mathrm{d}\tilde{C}_o}{\mathrm{dt}} = e_oB_o\left(C_o - \mu_o\tilde{C}_o\right) \tag{2}$$

Algorithm 1 Data fitting procedure for lymphocyte trafficking model

```
e, μ = get_initial_estimate_of_parameters()                          ▷ Using Equation 2.
e, μ, score = outer_loop(score, e, μ)
function OUTER_LOOP(outerLoopScore, e, μ)
    i=0
    score = outerLoopScore
    while i=0 or score ¡ outerLoopScore do
        outerLoopScore = score
        j=0
        while j==0 or score ¡ oldScore do
            oldScore = score; j++
            e, μ, score = improve_guess_with_analytics(e, μ)          ▷ Using Equation 3.
        end while
        j=0
        while j==0 or score ¡ oldScore do
            oldScore = score; j++
            e, μ, score = improve_worst_scoring_organ(e, μ)
        end while
        j=0
        while j==0 or score ¡ oldScore do
            oldScore = score; j++
            e, μ, score = improve_all_organs_sequentially(e, μ)       ▷ First organ 0, then organ 1, then...
        end while
        j=0
        while j==0 or score ¡ oldScore do
            oldScore = score; j++
            e, μ, score = improve_all_organs_emu_const(e, μ)          ▷ Fit each organ in turn with eμ held constant.
        end while
        j=0
        while j==0 or score ¡ oldScore do
            oldScore = score; j++
            e, μ, score = improve_high_flux_organ_against_others(e, μ)    ▷ Select the organ with the highest
    value of e_oB_o, and fit its parameters against other organs' (in turn or all at once).
        end while
        j=0
        while j==0 or score ¡ oldScore do
            oldScore = score; j++
            e, μ, score = improve_most_changed_parameters(e, μ)       ▷ Select the parameters that have been
    changed the most by this procedure, and fit them against each other.
        end while
        i++
    end while
    return e, μ, score
end function
```

Liver:

$$V_{\mathrm{liver}}\frac{\mathrm{d}C_{\mathrm{liver}}}{\mathrm{dt}} = B_{\mathrm{liver}}(C_{\mathrm{h}} - C_{\mathrm{liver}}) + e_{\mathrm{spleen}}\mu_{\mathrm{spleen}}B_{\mathrm{spleen}}(\tilde{C}_{\mathrm{spleen}} - C_{\mathrm{liver}}) + \sum_{o'\in\mathrm{mesentery}} B_{o'}(C_{o'} - C_{\mathrm{liver}})(1-e_{o'})$$

$$\begin{aligned}\tilde{V}_{\mathrm{liver}}\frac{\mathrm{d}\tilde{C}_{\mathrm{liver}}}{\mathrm{dt}} &= e_{\mathrm{liver}}B_{\mathrm{liver}}\left(C_{\mathrm{liver}} - \mu_{\mathrm{liver}}\tilde{C}_{\mathrm{liver}}\right)\\ &\quad + \sum_{o'\in\mathrm{mesentery}} e_{\mathrm{liver}}\left(C_{\mathrm{liver}} - \mu_{\mathrm{liver}}\tilde{C}_{\mathrm{liver}}\right)B_{o'}(1-e_{o'})\\ &\quad + e_{\mathrm{liver}}\left(C_{\mathrm{liver}} - \mu_{\mathrm{liver}}\tilde{C}_{\mathrm{liver}}\right)e_{\mathrm{spleen}}\mu_{\mathrm{spleen}}B_{\mathrm{spleen}},\end{aligned} \tag{3}$$

Lymph nodes:

$$\begin{aligned}V_{\mathrm{LN}}\frac{\mathrm{d}\tilde{C}_{\mathrm{LN}}}{\mathrm{dt}} =& e_{\mathrm{LN}}B_{\mathrm{LN}}\left(C_{\mathrm{LN}} - \mu_{\mathrm{LN}}\tilde{C}_{\mathrm{LN}}\right)\\ &+ \sum_{o'\notin\{\mathrm{LN,spleen,PC}\}} e_{o'}\mu_{o'}B_{o'}(\tilde{C}_{o'} - \mu_{\mathrm{LN}}\tilde{C}_{\mathrm{LN}})\\ &+ e_{\mathrm{liver}}\mu_{\mathrm{liver}}\left(e_{\mathrm{spleen}}\mu_{\mathrm{spleen}}B_{\mathrm{spleen}} + \sum_{o'\in\mathrm{mesentery}} B_{o'}(1-e_{o'})\right)(\tilde{C}_{\mathrm{liver}} - \mu_{\mathrm{LN}}\tilde{C}_{\mathrm{LN}}),\end{aligned} \tag{4}$$

Heart:

$$\begin{aligned}V_{\mathrm{h}}\frac{\mathrm{d}C_{\mathrm{h}}}{\mathrm{dt}} =& - C_{\mathrm{h}}\sum_{o} B_o\\ &+ \sum_{o\notin\mathrm{mesentery}} (1-e_o)B_oC_o\\ &+ \left(e_{\mathrm{spleen}}\mu_{\mathrm{spleen}}B_{\mathrm{spleen}} + \sum_{o\in\mathrm{mesentery}} (1-e_o)B_{\mathrm{o}}\right)(1-e_{\mathrm{liver}})C_{\mathrm{liver}}\\ &+ e_{\mathrm{LN}}\mu_{\mathrm{LN}}B_{\mathrm{LN}}\tilde{C}_{\mathrm{LN}} + \sum_{o\notin\{\mathrm{spleen,PC,LN}\}} \mu_{\mathrm{LN}}e_o\mu_oB_o\tilde{C}_{\mathrm{LN}}\\ &+ e_{\mathrm{liver}}\mu_{\mathrm{liver}}\mu_{\mathrm{LN}}\tilde{C}_{\mathrm{LN}}\left(e_{\mathrm{spleen}}\mu_{\mathrm{spleen}}B_{\mathrm{spleen}} + \sum_{o\in\mathrm{mesentery}} B_o\,(1-e_o)\right)\\ &+ e_{\mathrm{PC}}\mu_{\mathrm{PC}}B_{\mathrm{PC}}\tilde{C}_{\mathrm{PC}},\end{aligned} \tag{5}$$

Tumour invading lymphocytes:

$$\frac{\mathrm{d}N_{\mathrm{infiltrate}}}{\mathrm{dt}} = e_{\mathrm{tmr}}\frac{\tilde{V}_{\mathrm{tmr}}}{\tilde{V}_{\mathrm{TBO}}}B_{\mathrm{TBO}}C_{\mathrm{TBO}}, \tag{6}$$

The healthy region of the tumour bearing organ (TBO):

$$\tilde{V}_{\mathrm{TBO}}\frac{\mathrm{d}\tilde{C}_{\mathrm{TBO}}}{\mathrm{dt}} = e_{\mathrm{TBO}}\left(C_{\mathrm{TBO}} - \mu_{\mathrm{TBO}}\tilde{C}_{\mathrm{TBO}}\right)\left(1 - \frac{\tilde{V}_{\mathrm{tmr}}}{\tilde{V}_{\mathrm{TBO}}}\right)B_{\mathrm{TBO}}. \tag{7}$$

A.4 Model by Ganusov and Auerbach (2014)

We repeated our analyses on a model published by Ganusov and Auerbach [3], beginning with the generation of synthetic data from their best fit, rather than fitting to the original data, as the aim is to analyse identifiability.

The organs modelled are the lung, liver, spleen, subcutaneous lymph nodes, mesenteric lymph nodes and Peyer's patches. Equations for 'normal' compartments take the form,

$$\frac{\mathrm{d}x_i}{\mathrm{dt}} = m_i^{\mathrm{bld}} x_{\mathrm{bld}} - m_{\mathrm{bld}}^i x_i, \tag{8}$$

where x_i and x_{bld} are the proportional localisation in organ i and the blood, and m_j^i is the transfer rate from compartment i to compartment j. Several lymph node compartments in the model form a chain, such that,

$$\begin{aligned} \frac{\mathrm{d}x_{i1}}{\mathrm{dt}} &= m_i^{\mathrm{bld}} x_{\mathrm{bld}} - m_{\mathrm{bld}}^i x_{i1}, \\ \frac{\mathrm{d}x_{ij}}{\mathrm{dt}} &= m_{\mathrm{bld}}^i x_{i(j-1)} - m_{\mathrm{bld}}^i x_{ij} \qquad j = 2, \ \dots \ , k, \end{aligned} \tag{9}$$

where k is the number of sub-compartments for the given lymph node i. The final Peyer's patches sub-compartment is additionally assumed to drain into the first mesenteric lymph node sub-compartment. The blood compartment receives drainage from the final sub-compartment of all organs and loses cells (to decay or excretion) at a rate d. No other compartment is treated specially. Initial estimates for parameters can be obtained as in Section 2.1.3. Timescales associated with parameters are

$$\begin{aligned} \tau_i^{\mathrm{input}} &= \frac{1}{m_i^{\mathrm{bld}}} \\ \tau_i^{\mathrm{output}} &= \frac{1}{m_{\mathrm{bld}}^i}. \end{aligned} \tag{10}$$

Unlike the model in Section 2.1, they correspond to localisation in the same organ compartment, but their impact at short (input) and long (output) timescales on the data is expected to be the same.

A.5 Model by Singh *et al* (2020)

Finally, we analysed the trafficking section of the model published by Singh *et al* [4], originally published by Khot *et al* [5]. Again, we began by generating synthetic data from their best fit, rather than fitting to the original data. The equations are similar to those in Section 2.1. The equations for organs with no special anatomical features are,

$$\begin{aligned} V_o \frac{\mathrm{d}C_o}{\mathrm{dt}} &= B_o C_{\mathrm{Lung}}^V - J_o V_o C_o - (B_o - L_o) C_o, \\ \tilde{V}_o \frac{\mathrm{d}\tilde{C}_o}{\mathrm{dt}} &= J_o V_o C_o - L_o \tilde{C}_o, \end{aligned} \tag{11}$$

where many symbols have the same meanings as in Section 2.1, J_o is the transmigration rate of cells from the vasculature to the interstitial space of organ o and L_o is the lymph flow of organ o, defined as 0.02% of the blood flow ($L_o = 0.0002 B_o$) for every organ but the lymph nodes, whose lymph flow is equal to the sum of all other organ lymph flows. Note that with lymph flow fixed, the input J_o is the only non-anatomical parameter controlling localisation in each organ. Note also that $J_o = e_o B_o / V_o$, using parameter definitions from Section 2.1. J_o is not bounded; values used by Singh *et al* give values of e_o greater than 1.0. Similarly, values of J_o allow for cells to be created as they move between the vascular and interstitial compartments, which are subsequently destroyed as they leave the interstitial compartment, due to an imbalance of output and input flows equal to $L_o - J_o V_o$.

There are various anatomical differences between this system and that in Section 2.1. In Singh *et al*'s model, cells that drain to the heart first traverse through the pulmonary circuit before turning to organ vasculature. The spleen and pulmonary circuit have lymph flow, which drains to lymph nodes. The lymph nodes have no blood supply. Some members of the mesentery are missing, but those present still drain to the liver. Initial estimates for $\boldsymbol{J}$ can be obtained similarly to Section 2.1.3. The timescales associated with model parameters are,

$$\begin{aligned} \tau_o^{\mathrm{entry}} &= \frac{1}{J_o}, \\ \tau_o^{\mathrm{exit}} &= \frac{L_o}{\tilde{V}_o}, \end{aligned} \tag{12}$$

where the exit timescale is notably fixed to known anatomical parameters for each organ, due to the assumption that lymph flow rates are equal to 2% of blood flow rates.

B Appendix: Supplementary Results

B.1 Analysis of model by Ganusov and Auerbach (2014)

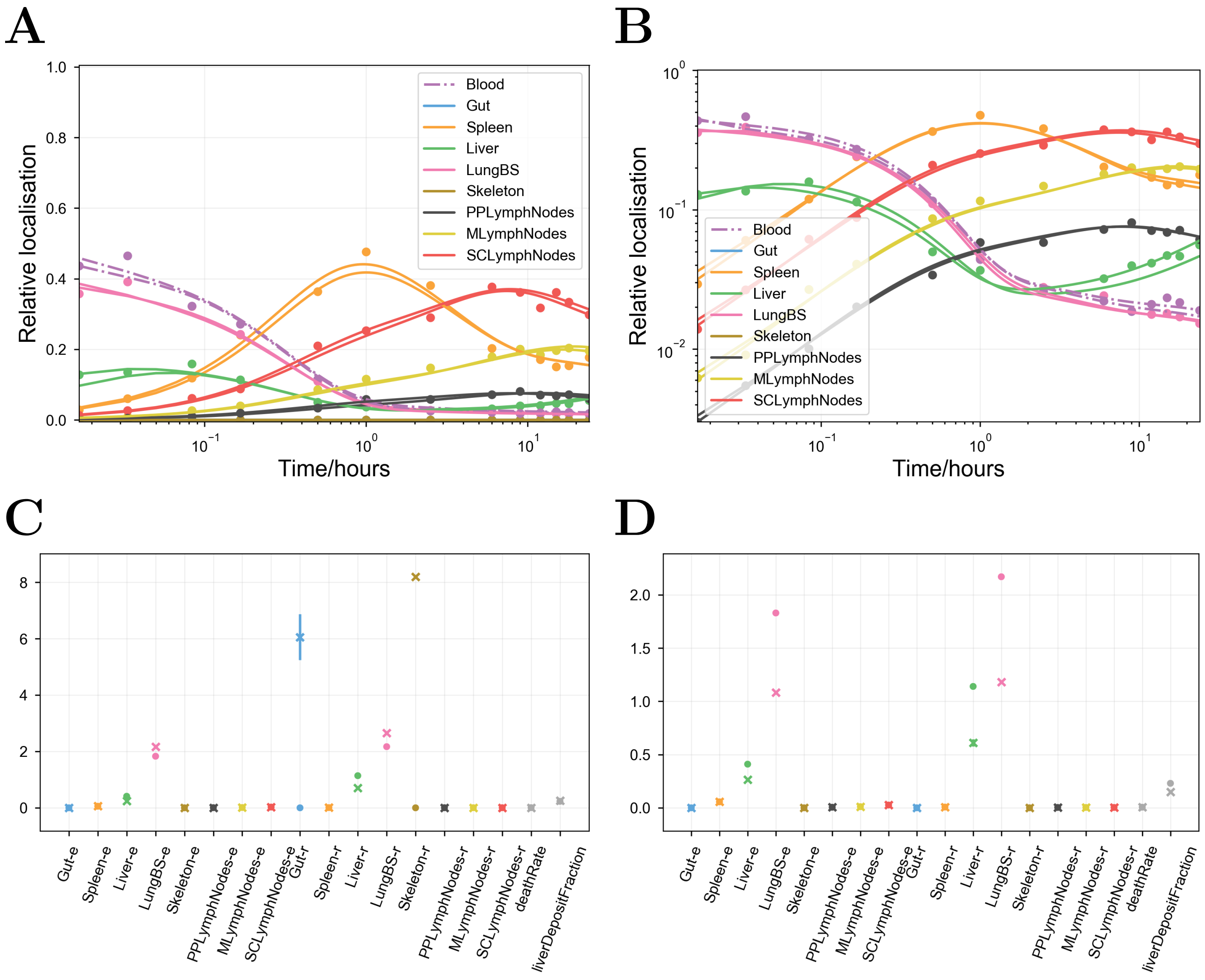

Figure S1: **(A, B)** Least-squares best fits of the ODE system published by Ganusov and Auerbach [3], Equation 8, to thirteen noisy synthetic data points plus initial conditions per organ generated from their published best fits, with the true solution plotted alongside. The noise was generated by multiplying all data by a Gaussian of mean 1 and standard deviation 0.1. The two fits are visually indistinguishable. Panel A is the result of plotting to unmodified data. Panel B is the result of plotting to the log of the data. **(C, D)** Least-squares best fit parameter values (crosses x) and the true values (circles o) for each fit. Lines show the local covariance of the fit score against parameter estimates.

We analysed least-squares fits of the model presented by Ganusov and Auerbach [3] to one of the data sets they presented, originally published by Smith and Ford [6]. We ran their model with their published parameters and used the model output to produce a synthetic dataset. We multiplied the data by Gaussian noise and fit their model to the noisy data. We then repeated the entire analysis, instead fitting the *log* of the model output to the log of the data. The results, shown in Figure S1, indicate the same problem as presented with our own model. When the least-squares best fits to the synthetic data and the true fits are plotted on the same axes, they are visually indistinguishable, but several parameter values are very different from their true values. The local

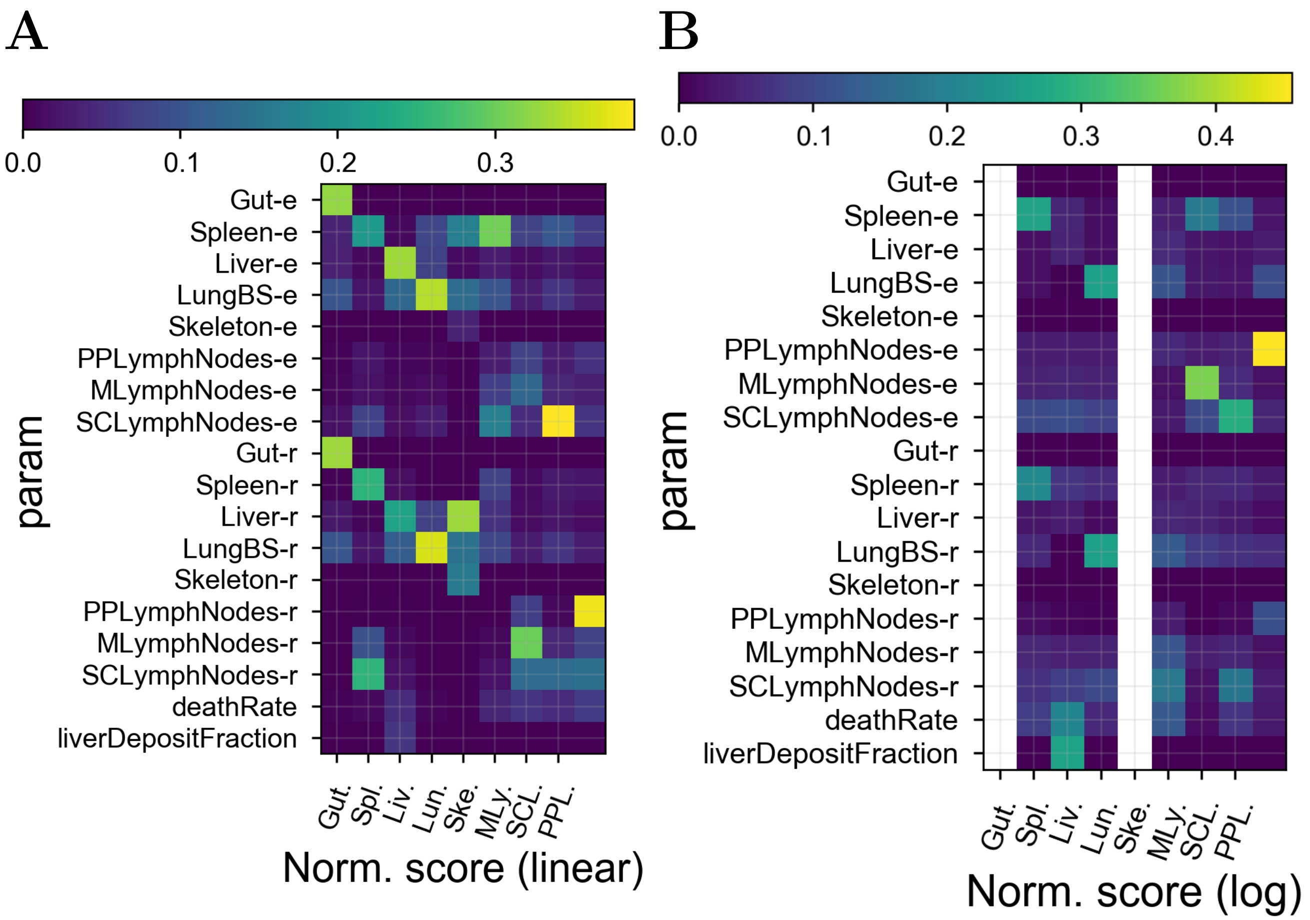

C

Name	Linear fit / true values		Eqbrm times/hours		Log fit / true values		Eqbrm times/hours	
	e	μ	Vasc.	Inst.	e	μ	Vasc.	Inst.
Gut	6.8e-15 / 0	6.06 / 0	2.4e+12	0.00275	0 / 0	6.9e-15 / 0	inf	1.3e+14
Spleen	1.07	1.20	0.277	1.99	1.04	1.02	0.286	2.34
Liver	0.59	0.62	0.0692	0.0236	0.65	0.54	0.0627	0.0273
LungBS	1.18	1.22	0.00769	0.00629	0.59	0.54	0.0154	0.0141
Skeleton	3.5e-06 / 0	8.199 / 0	4.8e+03	0.00203	0 / 0	3.4e-06 / 0	inf	5.8e+11
PPLymphNodes	0.79	0.81	7.95	12.1	1.01	0.93	6.23	10.6
MLymphNodes	0.93	0.91	3.39	10.7	1.03	0.91	3.06	10.8
SCLymphNodes	0.91	1.07	1.41	9.2	1.00	0.99	1.28	9.91
Death rate	0.87				1.21			
Liver deposit	1.13				0.65			

Figure S2: Local sensitivity analysis of the least-squares best fits to the synthetic data produced from Ganusov and Auerbach's fit, shown in Figure S1. **A)** Sensitivity of the fit to the data. **B)** Sensitivity of the fit to the log of the data. **C)** Parameter fits and timescales corresponding to the two displayed fits. Highlighted values are earlier than the first data point (1 minute) or greater than the final time point (24 hours). The sensitivity analysis was performed as described in Figure 6. The large or infinite timescales for some organs are due to the very small parameter values, see Equation 10.

sensitivity analysis, presented in Figure S2, indicate several parameters that may be expected to be fit inaccurately. As before, local sensitivity and timescale analyses implicate the parameters that were poorly fit, but the analyses also cast doubt on parameters that were, in fact, close to their true values.

B.2 Analysis of model by Singh *et al* (2020)

We analysed least-squares fits of the model presented by Singh *et al* [4] to the data that they published of the localisation of anti-EGFR CAR T-cells. We produced another synthetic dataset by running the model with their published parameters, with the exception of those relating to binding and unbinding of CARs from target antigen, as we could not replicate results unless the given binding/unbinding parameters were modified. The predicted localisation of the number of CARs in the tumour compartment is therefore lower than Singh *et al* reported. We multiplied the synthetic data by Gaussian noise of mean 1 and standard deviation σ, as before, and attempted to recover the original parameters. In this instance, no blood data was provided, making it harder to fit the order of magnitude of the transmigration rates into organs, J. If the relative values of J are held constant, their mean value can be changed without greatly impacting the fit until the timescale of the entry of cells into organs is on the timescale of the data. This results in considerably degeneracy in fitted parameter values. The first attempt to fit parameter values were made with initial values far from the true values, resulting in the fit converging to a local minimum. To remedy this, the parameters that Singh *et al* fixed for organs that lacked data (the gut, lungs, brain and miscellaneous organs) were fixed to the same values, and other parameters given initial values of a similar order of magnitude. This resulted in fits that more reliably reproduced Singh *et al*'s parameter values.

Unlike the models analysed in Sections 3.4 and B.1, fits to synthetic data produced by Singh *et al*'s model could reproduce the correct parameters with Gaussian noise of standard deviation 0.1 and even 0.15 (data not shown). Noise with a standard deviation of 0.2 produced results similar to those seen with the other models; the best fit to this noisy data was qualitatively similar to the true fit but the parameter for the spleen is considerably different from the true value. The sensitivity analysis indicates that only the tumour invading lymphocytes (TILs), liver and death rate parameters might be expected to be accurate. The greatest influence on all data actually comes from the transmigration rate to miscellaneous organs, though this is a fixed, non-zero value obtained from the literature. As noted in the discussion section, this model was more robust to noise than the other two, but this result may have been a consequence of fixing the output rate of cells from all organs and the input rates to several organs (gut, lung, brain and miscellaneous organs). Timescales were calculated for the input and output rates into each organ o using Equation 12 and are shown in Figure S3D. Input timescales are all less than the first data time point (2 days) except for the brain and skin, and output timescales are all less than the final time point except for the skin. If this were the case for the first model presented, Equation 1, we might expect that all parameters would be incorrectly fit. In that model, one parameter for each organ (μ_o) determined the steady state localisation in each organ, and one (e_o) determined the timescale on which steady state is reached. In this model, there is only one parameter per organ, which determines steady-state localisation. The parameter should be expected to be fit correctly as long as the longest of the two timescales is on the scale of data time-points. However, the spleen is poorly fit despite both its input and output timescales being short compared to data. The reason for this is that the spleen's output connects to the liver. The liver is the organ with the greatest (absolute) localisation and the slowest exit timescale, so it has a large impact on the localisation in all organs. As spleen equilibrium depends on the rate at which cells leave the liver *and* the spleen, the spleen parameter is relatively insensitive compared to the liver parameter.

B.3 Bayesian computation

As in Section 3.5, a Markov Chain Monte Carlo technique was used to fit the system by Singh *et al* to synthetic data. As before, three Markov chains, a burn-in of 1000 samples and repeated cycles of 5000 iterations per chain were used. R_{hat} (measure of how the interchain variation compares to the intra-chain variation) was below the cut-off threshold of 1.1 after a total of 75000 samples per chain. This is fewer samples than for the trafficking ODEs, likely because this system has fewer parameters. Results are shown in Figure S4. Panel A shows true parameter values (used to generate the synthetic data) against best fit parameters and their standard deviations, calculated from the final parameter values in the three Markov chains. Unlike in previous plots, these standard deviations typically do overlap the true values. Panel B shows a density plot of parameter values in the last 5000 iterations of each Markov chain (15,000 parameter sets in total), showing that some parameter densities are highly peaked (indicating a good fit) and others are not (indicating an unidentifiable or poorly fit parameter). Panels C through E show the density

A B

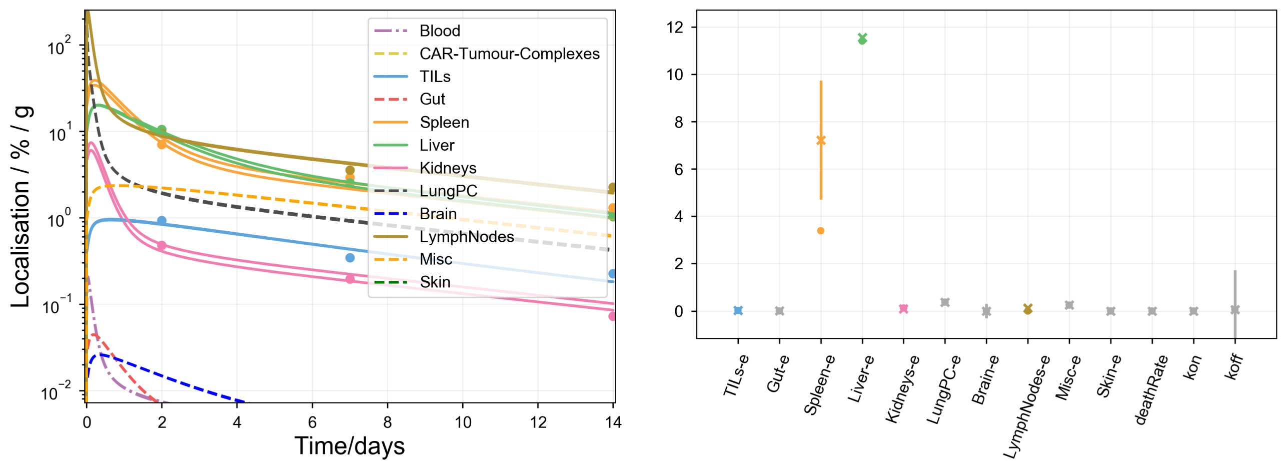

C D

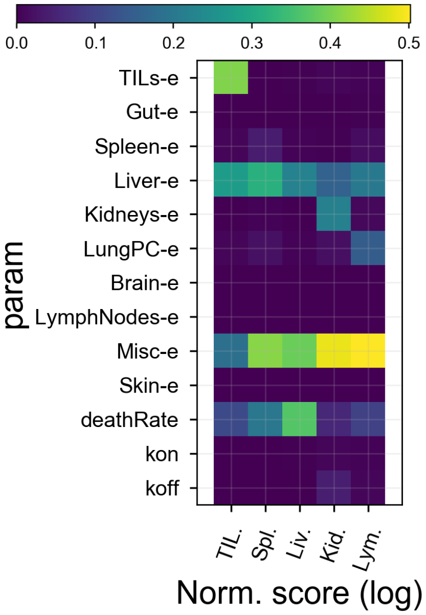

Name	e / true value	Eqbrm times	
		Vasc.	Inst.
Tumour	1.0	7.08 hours	3.6 days
Gut	1.0	6.29 hours	12.0 hours
Spleen	2.1	0.38 mins	15.5 hours
Liver	1.0	0.11 mins	7.79 days
Kidneys	0.8	3.34 mins	5.76 hours
Lung PC	1.0	0.16 mins	12.3 mins
Brain	1.0	2.63 days	1.54 days
L. nodes	0.1184/0.0	18.2 mins	18.2 mins
Misc	1.0	0.47 mins	4.48 days
Skin	0.0/0.0	inf	inf

Figure S3: **(A)** Least-squares best fits of the ODE system published by Singh *et al* [4], Equation 11, to three noisy synthetic data points plus initial conditions per organ generated from their published best fits, with the true solution plotted alongside. The noise was generated by multiplying all data by a Gaussian of mean 1 and standard deviation 0.2. The two fits are visually indistinguishable. Solid lines show outputs for which data is present. Dashes lines show output from modelled organs for which no data is present. **(B)** Least-squares best fit parameter values (crosses x) and the true values (circles o) for each fit. Lines show the local covariance of the fit score against parameter estimates. **(C)** Local sensitivity analysis at the least-squares best fit parameters. **(D)** The timescale associated with each parameter value.

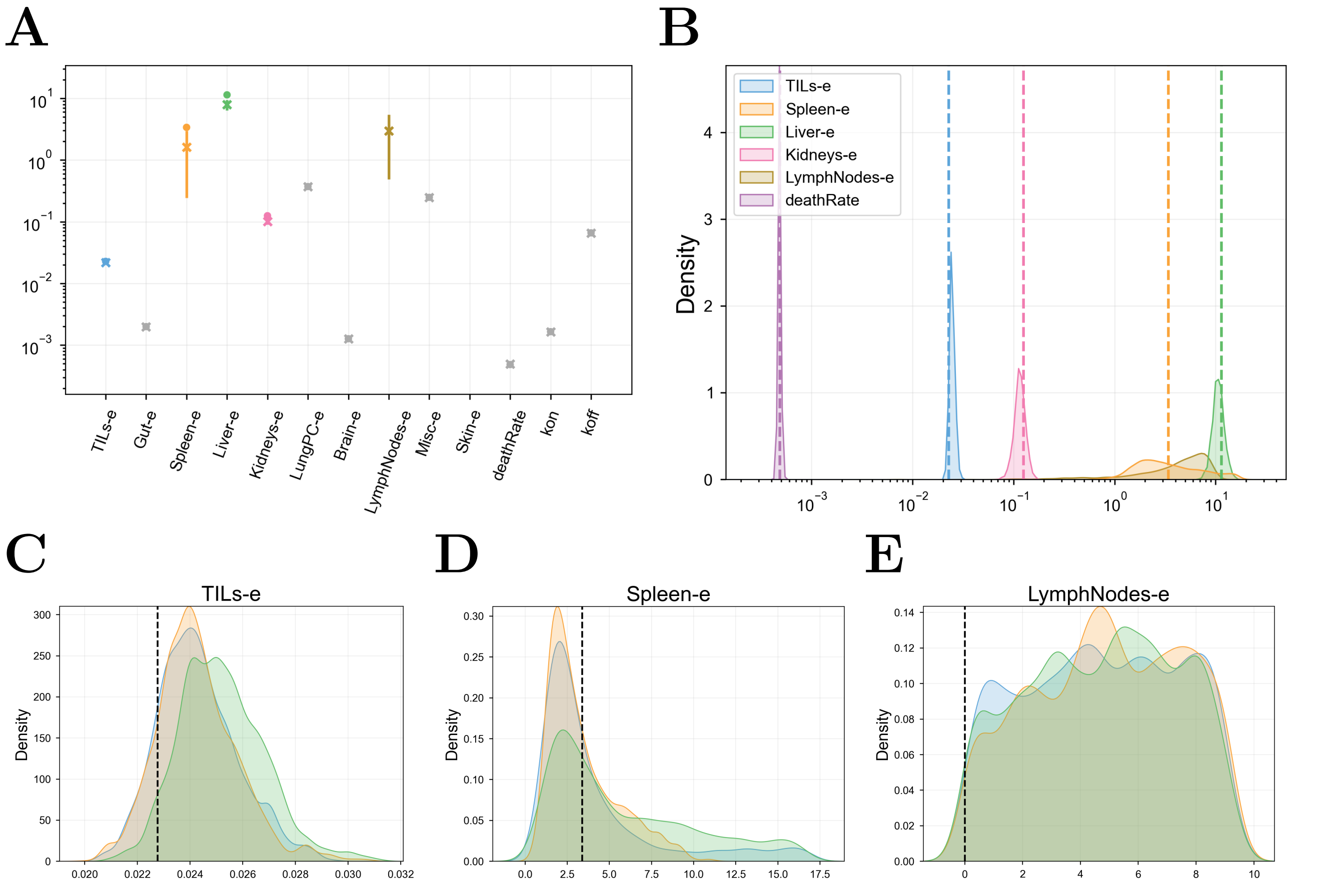

Figure S4: The distribution of parameters produced by fitting the ODE system published by Singh *et al* [4], Equation 11, to three noisy synthetic data points plus initial conditions per organ generated from their published best fits. Fits were made through adaptive covariance Monte Carlo [7]. **A)** Best fit parameter values (crosses x) and the true values (circles o) for each fit. Standard deviations (vertical lines) are calculated across the final value of the three Markov chains. Grey-coloured parameters are fixed to values published by Singh *et al.* **B)** Density plot of parameter values across the last 5000 samples of every Markov chain. Dashed lines indicate true values of parameters. There is no line for lymph nodes because it would be positioned at 0.0. **C-E)** Density plots of the values of particular parameters in the last 5000 samples of each Markov chain in turn. Dashed lines indicate true values of parameters.

plots from the last 5000 samples of each chain separately, for the tumour (panel C), spleen (panel D) and lymph node (panel E) parameters. The tumour parameter is well-fit and the spleen parameter is poorly fit, but the lymph node parameter is completely unidentifiable.

References

[1] Mario Castro and Rob J. de Boer. Testing structural identifiability by a simple scaling method. *PLoS Comput. Biol.*, 16(11):e1008248, Nov 2020. ISSN 1553-7358. doi: 10.1371/journal.pcbi.1008248.

[2] Liam V. Brown, Eamonn A. Gaffney, Ann Ager, Jonathan Wagg, and Mark C. Coles. Quantifying the limits of CAR T-cell delivery in mice and men. *J. R. Soc. Interface*, 18(176):20201013, Mar 2021. ISSN 1742-5662. doi: 10.1098/rsif.2020.1013.

[3] Vitaly V. Ganusov and Jeremy Auerbach. Mathematical Modeling Reveals Kinetics of Lymphocyte Recirculation in the Whole Organism. *PLoS Comput. Biol.*, 10(5):e1003586, May 2014. ISSN 1553-7358. doi: 10.1371/journal.pcbi.1003586.

[4] Aman P. Singh, Xirong Zheng, Xiefan Lin-Schmidt, Wenbo Chen, Thomas J. Carpenter, Alice Zong, Weirong Wang, and Donald L. Heald. Development of a quantitative relationship between CAR-affinity, antigen abundance, tumor cell depletion and CAR-T cell expansion using a multiscale systems PK-PD model. *mAbs*, 12(1):1688616, Jan 2020. ISSN 1942-0862. doi: 10.1080/19420862.2019.1688616.

[5] Antari Khot, Satoko Matsueda, Veena A. Thomas, Richard C. Koya, and Dhaval K. Shah. Measurement and Quantitative Characterization of Whole-Body Pharmacokinetics of Exogenously Administered T Cells in Mice. *J. Pharmacol. Exp. Ther.*, 368(3):503–513, Mar 2019. ISSN 0022-3565. doi: 10.1124/jpet.118.252858.

[6] M E Smith and W L Ford. The recirculating lymphocyte pool of the rat: a systematic description of the migratory behaviour of recirculating lymphocytes. *Immunology*, 49(1):83–94, 1983. ISSN 0019-2805.

[7] Michael Clerx, Martin Robinson, Ben Lambert, Chon Lok Lei, Sanmitra Ghosh, Gary R. Mirams, and David J. Gavaghan. Probabilistic Inference on Noisy Time Series (PINTS). *J. Open Res. Software*, 7(1), Jul 2019. ISSN 2049-9647. doi: 10.5334/jors.252.
